# Using RNA-seq to identify suitable housekeeping genes for hypoxia studies in human adipose-derived stem cells

**DOI:** 10.1186/s12860-023-00475-4

**Published:** 2023-04-17

**Authors:** Huan Ting Ong, Cecilia M. Prêle, Rodney J. Dilley

**Affiliations:** 1 Ear Science Institute Australia, Nedlands, Western Australia Australia; 2 Ear Sciences Centre, The University of Western Australia, Nedlands, Western Australia Australia; 3 Institute for Respiratory Health, The University of Western Australia, Nedlands, Western Australia Australia; 4 Discipline of Medical, Molecular and Forensic Sciences, Murdoch University, Murdoch, Western Australia Australia; 5 Centre for Cell Therapy and Regenerative Medicine, The University of Western Australia, Nedlands, Western Australia Australia

**Keywords:** Reference genes, qRT-PCR, RNAseq, Hypoxia, Human adipose-derived stem cells, ADSC

## Abstract

**Background:**

Hypoxic culture conditions have been used to study the impact of oxygen deprivation has on gene expression in a number of disease models. However, hypoxia response elements present in the promoter regions of some commonly used housekeeping genes, such as *GAPDH* and *PGK1,* can confound the relative gene expression analysis. Thus, there is ongoing debate as to which housekeeping gene is appropriate for studies investigating hypoxia-induced cell responses. Specifically, there is still contradicting information for which housekeeping genes are stable in hypoxia cultures of mesenchymal stem cells. In this study, candidate housekeeping genes curated from the literature were matched to RNAseq data of normoxic and hypoxic human adipose-derived stem cell cultures to determine if gene expression was modulated by hypoxia or not. Expression levels of selected candidates were used to calculate coefficient of variation. Then, accounting for the mean coefficient of variation, and normalised log twofold change, genes were ranked and shortlisted, before validating with qRT-PCR. Housekeeping gene suitability were then determined using GeNorm, NormFinder, BestKeeper, comparative$\Delta Ct$, RefFinder, and the Livak method.

**Results:**

Gene expression levels of 78 candidate genes identified in the literature were analysed in the RNAseq dataset generated from hADSC cultured under Nx and Hx conditions. From the dataset, 15 candidates with coefficient of variation ≤ 0.15 were identified, where differential expression analysis results further shortlisted 8 genes with least variation in expression levels. The top 4 housekeeping gene candidates, *ALAS1*, *RRP1*, *GUSB*, and *POLR2B*, were chosen for qRT-PCR validation. Additionally, *18S*, a ribosomal RNA commonly used as housekeeping gene but not detected in the RNAseq method, was added to the list of housekeeping gene candidates to validate. From qRT-PCR results, *18S* and *RRP1* were determined to be stably expressed in cells cultured under hypoxic conditions.

**Conclusions:**

We have demonstrated that *18S* and *RRP1* are suitable housekeeping genes for use in hypoxia studies with human adipose-derived stem cell and should be used in combination. Additionally, these data shown that the commonly used *GAPDH* and *PGK1* are not suitable housekeeping genes for investigations into the effect of hypoxia in human adipose-derived stem cell.

**Supplementary Information:**

The online version contains supplementary material available at 10.1186/s12860-023-00475-4.

## Background

Housekeeping genes (HKG) are stable, constitutively expressed reference genes which are used to normalise the expression of genes-of-interest in a variety of molecular techniques. HKG need to be ubiquitously and stably expressed in order to accurately quantify any treatment-related differential gene expression. For this purpose, it is usual to select HKG involved in supporting cellular functions [1]. Typically, HKG are selected from related literature, or most commonly determined from previous work performed in the same laboratory, sometimes without sufficient regard to the stability of that HKG to the different treatments being applied. The stability of HKG expression should be examined for each experimental design, and specific cell types [2], as it cannot be assumed that a “one-size-fits-all” approach is suitable.

Hypoxic (Hx) culture conditions have been used to model cellular physiological responses in various ischemic disorders, such as ischemic brain injury [3, 4], tumour progression and hypoxic microenvironment [5, 6], diabetes and obesity [7, 8], and toxin-induced reactive oxygen species in chronic kidney diseases [9]. The molecular response to hypoxia is typically driven by hypoxia response elements (HRE) present in the promoter region of genes. Functional HRE contain a target *cis*-acting DNA sequence that binds hypoxia-inducible factor -1 (HIF-1) protein complex which regulates gene expression [10, 11]. As hypoxia is a fundamental change to culture conditions, other genes regulated by hypoxia can be involved in metabolic switching pathways such as redox homeostasis and glycolysis, hence a large number of complex interactions make it difficult to select for HKG based on the absence of HRE alone. A number of commonly used HKG contain HRE and are regulated by hypoxia, for example *GAPDH* [12–14] and *PGK1* [10]. A list of 114 genes which are induced by hypoxia is shown in Additional file 1 [15, 16]).

While various HKG have been reported as suitable for hypoxia studies (summarised in Table 1), there is ongoing debate in the literature as to which HKG are stably expressed in hypoxia [17–27], especially in mesenchymal stem cells [20, 28–30]. Thus, using a standard protocol for identifying multiple reference genes, we selected *RPL13A*, *TBP* and *GAPDH* from the literature and performed a preliminary qRT-PCR to study gene regulation in Hx human adipose-derived stem cells (hADSC), but found that these genes, unlike what they’ve been reported, were not stably expressed (Additional file 2). This represents a major setback which requires an alternative solution.

**Table 1:** Literature reported HKG suitable for hypoxia studies

Gene Name	Gene Symbol	Reference
18S rRNA	*18S*	[17]
beta-actin	*ACTB*	[18, 19]
ribosomal protein lateral stalk subunit P0	*RPLP0*	[18]
beta-glucuronidase	*GUSB*	[18, 20]
transferrin receptor	*TFRC*	[18]
beta-2-microglobulin	*B2M*	[21, 22]
ribosomal protein L13a	*RPL13A*	[21, 23]
RNA polymerase II	*RPII*	[21]
28S rRNA	*28S*	[17, 24]
hypoxanthine phosphoribosyltransferase 1	*HPRT1*	[23, 25]
tyrosine 3/tryptophan 5-monooxygenase activation protein	*YWHAZ*	[20]
TATA-box binding protein	*TBP*	[20, 22]
glyceraldehyde 3-phosphate dehydrogenase	*GAPDH*	[19, 26, 28–30]
peptidylprolyl isomerase A	*PPIA*	[27]

Alternative to testing potential HKG from the literature one at a time, approaches have been developed to identify and quantify a panel of selected HKG candidates based on the gene expression values from qRT-PCR data, such as GeNorm [31] which uses a geometric averaging method, NormFinder [32] a model-based variance estimation method, BestKeeper [33] a pair-wise comparison method, comparative $\Delta Ct$ method [34] by relative expression, and RefFinder [35] which uses a geometrically averaging weighted ranks for the listed methods above. However, these approaches require numerous qRT-PCR experiments with a set of at least 12 HKG candidates and involve a larger number of samples and reagents.

With the advancement of next generation sequencing technology, RNA sequencing (RNAseq) is an attractive tool that can be used to obtain a broad comparison between transcriptome profiles [1]. Some recent studies have used RNAseq datasets to select stable HKG [36–38] customized for a unique experimental design. Lemma et al. 2018 [37] selected HKG from RNAseq data using normalised log2 fold change (L2FC), while Tang et al. 2017 [36] and Wang et al. 2019 [38] normalised for coefficient of variation of mRNA transcript expressions when selecting for HKG. Due to the complex nature of gene expression and regulation by hypoxia, RNAseq may be an effective way to screen large transcriptome data for HKG selection.

In this study, we aim to use RNAseq data from hADSC to screen HKG from the literature and validate their expressions under hypoxic regulation, in order to identify the most suitable HKG in Hx hADSC. RNAseq datasets were generated from hADSC from 5 donors and cultured under Nx or Hx conditions in vitro*.* RNAseq and bioinformatic analysis was used to identify differences in the transcriptome between donors and culture conditions. Specifically, the stability of candidate HKG in cells cultured under Nx and Hx conditions was determined by calculating the coefficient of variation (CV) of both normalised and transformed read counts, and L2FC obtained from differential gene expression. Potential HKG candidates were identified by low CV (≤ 0.15), ranked and were validated using qRT-PCR where relative expression of each selected HKG was calculated using the ${2}^{-\Delta \Delta Ct}$ Livak method [39], GeNorm method, NormFinder method, the BestKeeper method, $\Delta Ct$ method and RefFinder method.

## Results

### Differential gene expression analysis

**Fig. 1:**
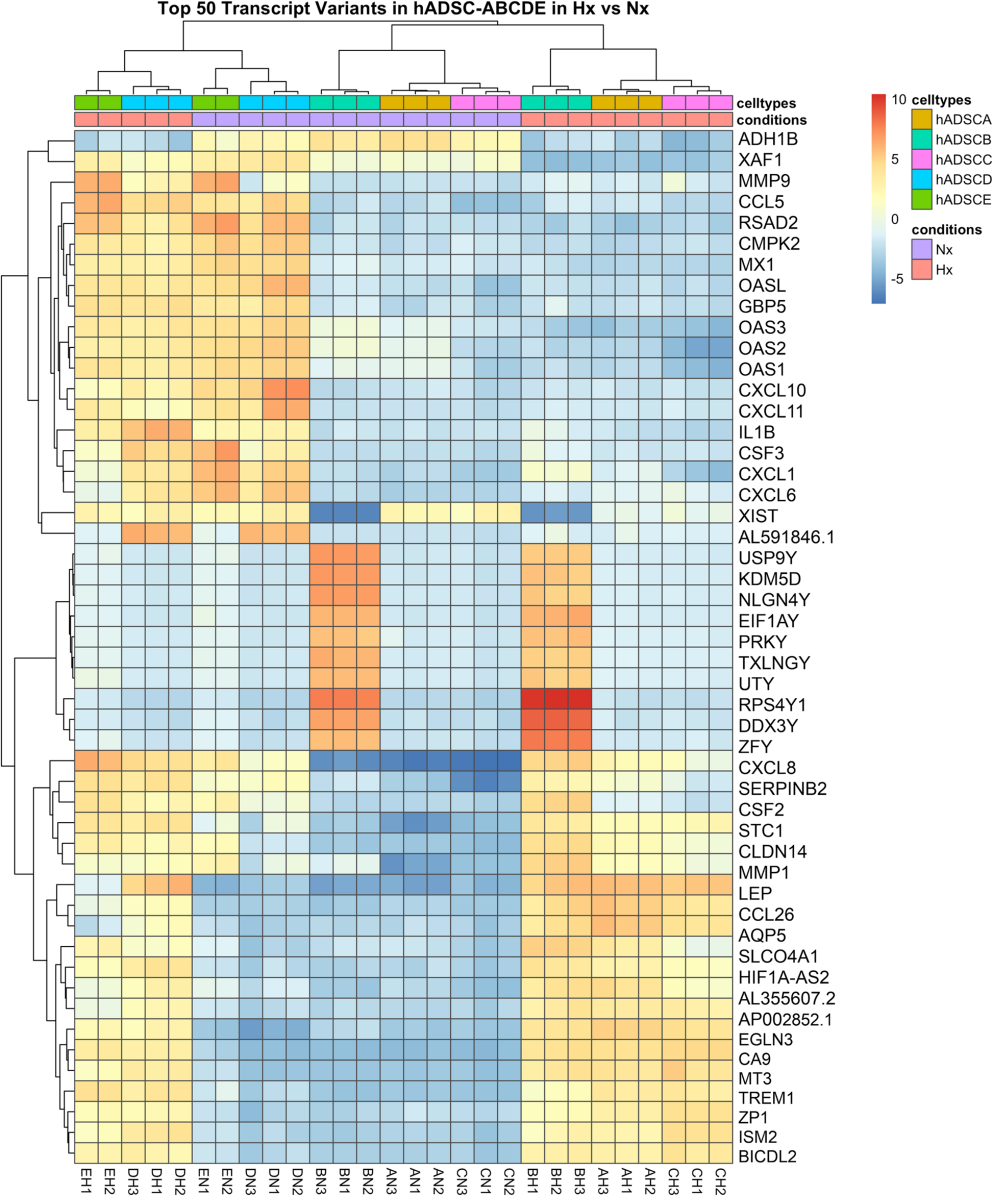
Heatmap for top 50 transcripts differentially expressed in hADSC under Hx and Nx. Normalised expression of the top 50 transcript variants from 5 human adipose-derived stem cell lines in hypoxic and normoxic conditions

**Fig. 2:**
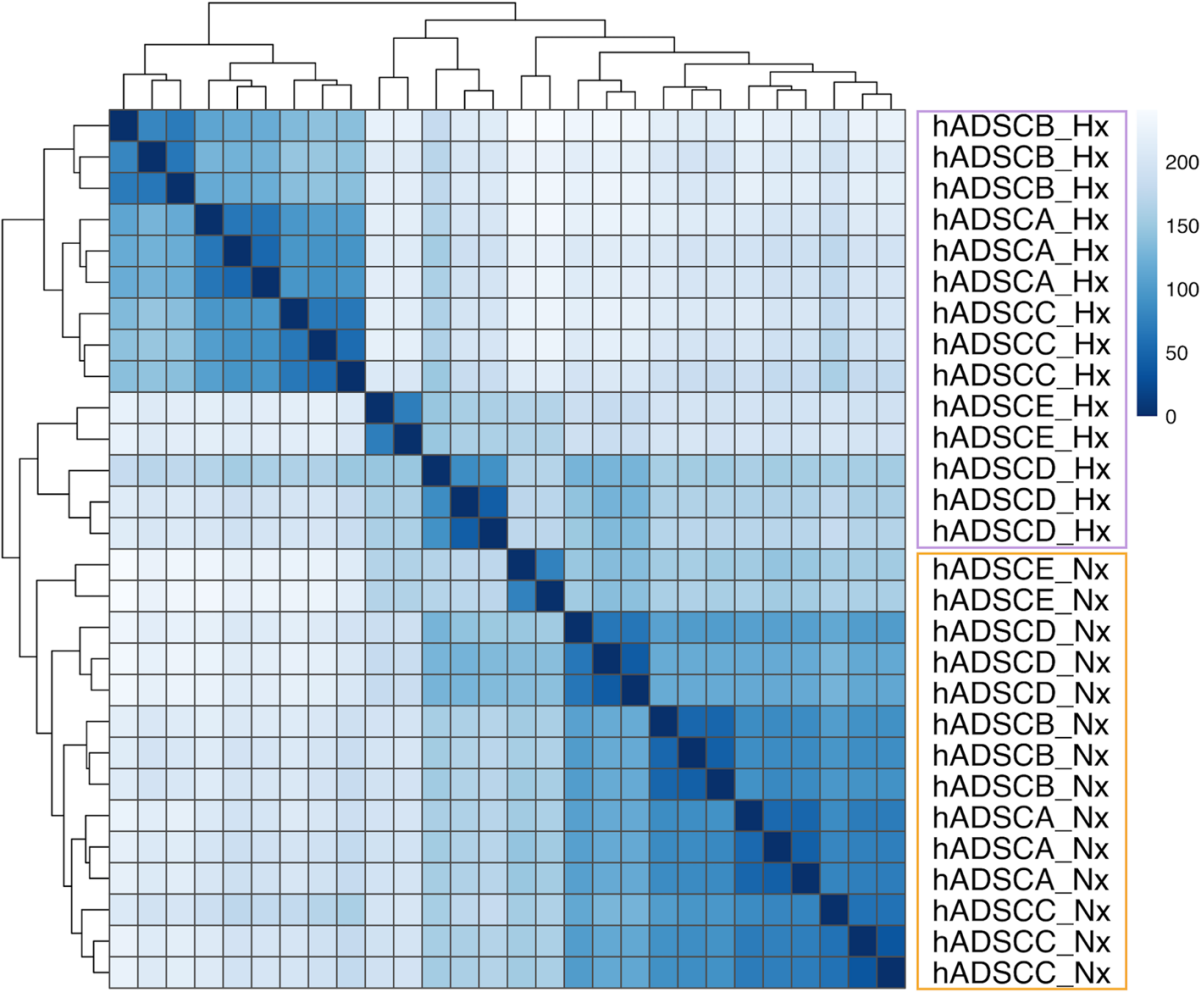
Sample-to-sample distances between hADSC lines. The sample-to-sample distances were plotted as a heatmap to show that greatest clustering occurred within hypoxic (Hx, purple) and normoxic (Nx, orange) treatment groups, followed by individual hADSC lines and then replicates

**Fig. 3:**
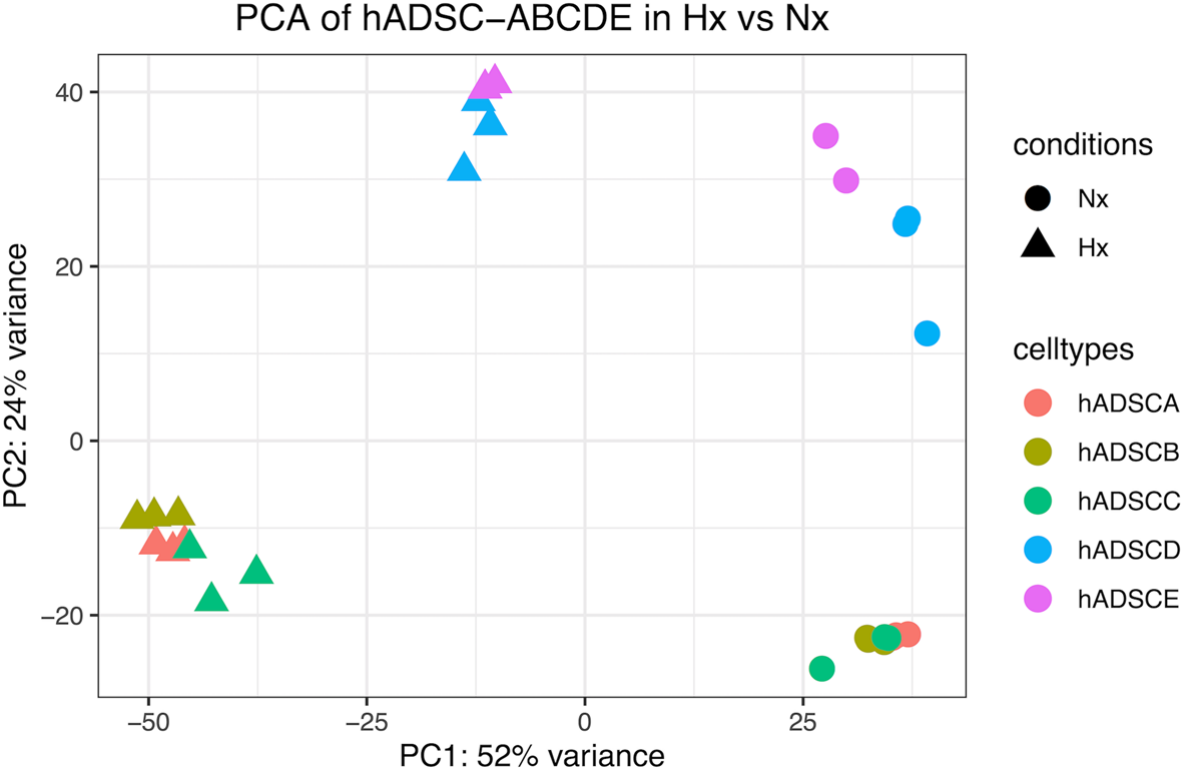
Principal Component Analysis (PCA) of hADSC datasets. PCA plot of hADSC lines (**A-E**, colour-coded) in hypoxic (Hx, triangles) and normoxic (Nx, circles) treatment groups. The first principal component (PC1) represents the maximum variance direction of data distribution at 52%, and the second principal component (PC2) represents the second largest variation at 24%

To identify which genes were differentially expressed by hADSC cultured under Hx conditions, the RNAseq transcriptional profile of five primary hADSC cell cultures (hADSC-A, -B, -C, -D, -E) grown under Hx conditions were compared to the profile of hADSC cells cultured under standard conditions (Nx). Analysis of the transcriptome revealed a total of 2800 differentially expressed transcripts; Fig. 1 shows the top 50 transcripts of at least |2| L2FC. DESeq2 was also used to cluster each dataset according to the similarities in transcript count distribution (Fig. 2). Principal Component Analysis (PCA, Fig. 3) identified distinct clusters of differentially expressed genes in hADSC cultured in Nx and Hx conditions, as well as a donor-to-donor variability in transcriptome profile between the hADSC lines.

### Comparison of hypoxic effects on HKG identified from the literature

**Fig. 4:**
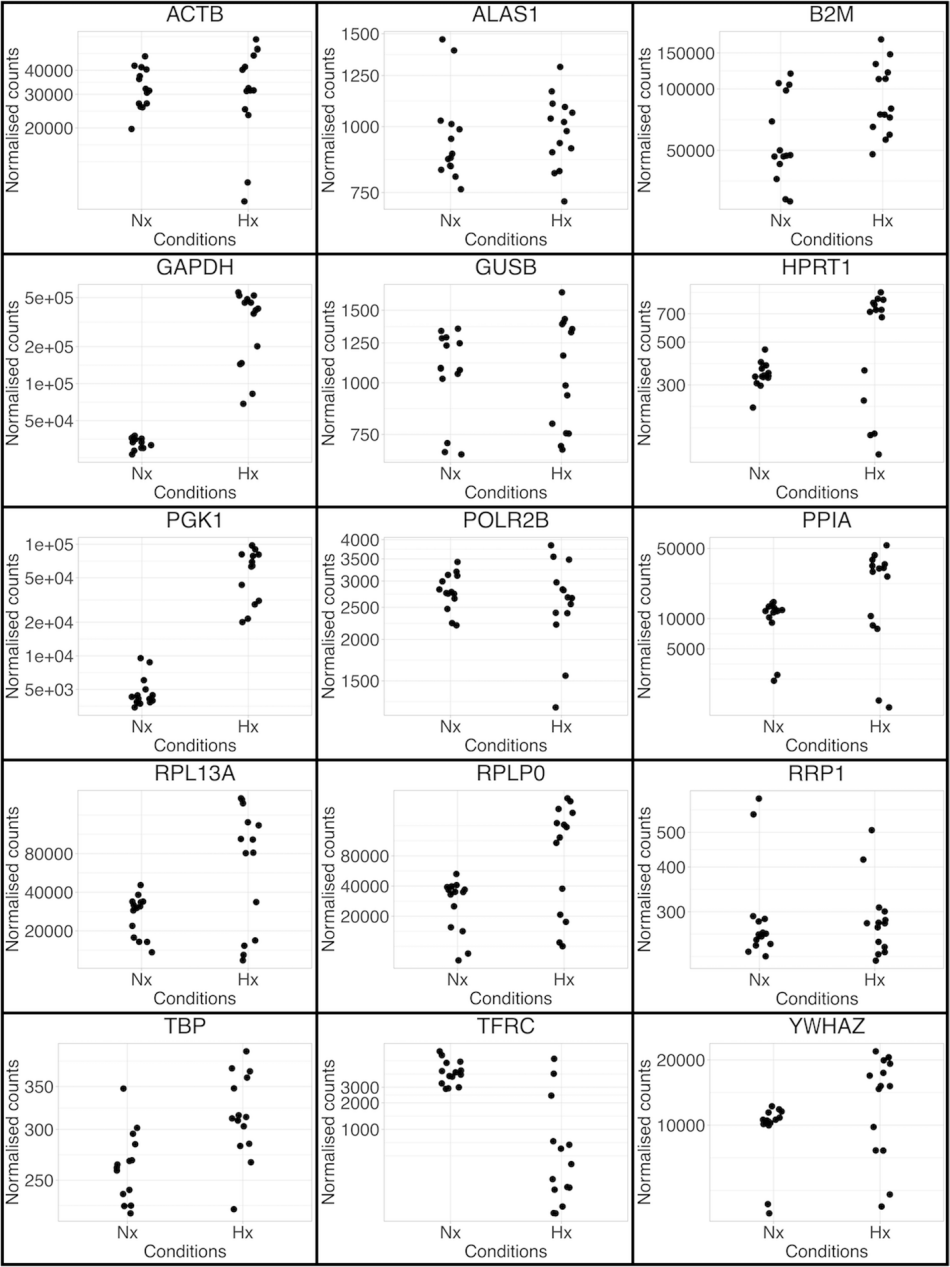
Distribution of counts for normalised expression of selected housekeeping genes. Normalised counts from the differential expression analysis by RNAseq of 5 hADSC donor lines in either Hx or Nx conditions are plotted for 15 selected housekeeping gene candidates

Candidate HKG were identified from the literature (*ACTB*, *ALAS1*, *B2M*, *GAPDH*, *GUSB*, *HPRT1*, *PGK1*, *POLR2B*, *PPIA*, *RPL13A*, *RPLPO*, *RRP1*, *TBP*, *TFRC* and *YWHAZ*) and matched for normalised gene expression in the RNAseq dataset generated from hADSCs cultured under Hx and Nx conditions (Fig. 4). Normalised counts indicated a similar distribution between Hx and Nx datasets for 7 of the candidates (*ACTB*, *ALAS1*, *B2M*, *GUSB*, *POLR2B*, *RRP1* and *TBP*). In contrast, another 7 genes (*GAPDH*, *HPRT1*, *PGK1*, *PPIA*, *RPL13A*, *RPLP0* and *YWHAZ*) increased expression in Hx samples compared to Nx hADSC, and *TFRC* showed reduced expression in Hx hADSC.

### Selection of HKG from the RNAseq dataset

To compare the levels of gene expression, data was first expressed in transcripts per million (TPM) normalised counts and DESeq2-normalised counts for the selected HKG. Both TPM and DESeq2-normalised counts were then used to calculate for CV. before the list of HKG were ranked smallest to largest for both CV and L2FC (Additional file 3). Then, the average CV was calculated from HKG with CV ≤ 0.15, before normalising CV and L2FC to the smallest corresponding values of the 78 candidates and summed. HKG were ranked from smallest to the largest value for the sum of these four methods. Table 2 shows the top 8 (least variation) HKG candidates calculated and the top 4 listed in Table 3 for validation with qRT-PCR, with data analysis workflow summarised in Additional file 4.

**Table 2:** Housekeeping gene candidates – selected on smallest coefficient of variance in TPM and DESeq2-normalised counts

Gene	Norm	CV calculated					L2FC	$\underline{CV}$	Norm CV	Norm L2FC	Arbitrary Ranking	
		A	B	C	D	E					Sum	Rank
*GUSB*	TPM	0.19	0.37	**0.11**	**0.04**	0.27	0.01^a^	0.01	1.1	1.0^a^	2.1	1
	DE	**0.09**	**0.08**	**0.14**	**0.04**	0.28						
*RRP1*	TPM	0.30	0.30	0.08	**0.13**	**0.15**	-0.02	0.02	1.2	2.2	3.4	2
	DE	**0.08**	**0.08**	**0.09**	**0.10**	0.16						
*ALAS1*	TPM	0.15	0.35	0.18	**0.03**	**0.14**	0.05	0.01^a^	1.0^a^	6.4	7.4	3
	DE	**0.11**	**0.08**	**0.06**	**0.06**	**0.14**						
*POLR2B*	TPM	**0.12**	0.33	0.27	**0.06**	0.38	-0.12	0.01	1.1	14.0	15.1	4
	DE	**0.13**	**0.05**	**0.12**	**0.05**	0.38						
*CHFR*	TPM	0.24	**0.15**	0.23	**0.13**	**0.12**	0.12	0.01	1.2	14.3	15.5	5
	DE	**0.05**	0.30	**0.10**	**0.08**	**0.11**						
*GGA1*	TPM	0.42	0.47	**0.11**	**0.15**	**0.14**	-0.15	0.02	1.8	17.9	19.7	6
	DE	0.20	0.16	**0.13**	**0.15**	**0.14**						
*YWHAZ*	TPM	**0.06**	**0.05**	**0.07**	0.22	**0.08**	0.39	0.01	1.1	47.2	48.2	7
	DE	0.28	0.34	0.18	0.18	**0.08**						
*NONO*	TPM	0.06	0.06	0.11	0.09	0.14	0.46	0.02	1.2	55.2	56.4	8
	DE	0.23	0.36	0.19	0.13	0.15						

Coefficient of variance was calculated from TPM and DESeq2-normalised values with L2FC values extracted from DESeq2. The average CV was calculated from HKG with CV ≤ 0.15 (bolded), before normalising CV and L2FC to the smallest corresponding values (^a^) of the 78 candidates and summed. HKG were ranked from smallest to the largest value for the sum of these four methods

**Table 3:** qRT-PCR analysis for selected housekeeping gene candidates in hypoxic and normoxic hADSC cultures

hADSC	Repeats	*ALAS1*		*RRP1*		*18S*		*PGK1*		*GUSB*	
		Hx	Nx	Hx	Nx	Hx	Nx	Hx	Nx	Hx	Nx
A	1	27.2	25.0	29.1	29.2	21.9	20.3	21.5	25.4		
		27.8	24.9	29.7	29.6	21.7	20.1	21.5	25.4		
		28.0	25.1	29.9	29.6	21.5	20.2	21.7	25.6		
	2	29.6	26.3	29.8	30.5					> 35	27.5
		29.5	26.1	29.6	30.4					> 35	27.3
		29.9	26.2	29.5	30.5					> 35	27.3
B	1	24.9	23.9	25.6	25.7	18.7	20.7	20.1	22.0		
		25.0	23.9	25.6	25.7	18.6	20.6	20.1	22.0		
		25.0	24.0	25.8	26.1	18.8	20.5	20.1	21.9		
	2	29.2	28.4	30.8	29.8					> 35	> 35
		28.9	28.1	30.5	29.7					> 35	> 35
		29.1	28.0	30.8	29.7					> 35	> 35
C	1	26.5	25.9	31.3	31.5	19.5	19.4	22.4	25.7	27.1	26.3
		26.3	25.8	31.1	31.2	19.7	19.3	22.4	25.7	27.1	26.3
		26.4	25.7	31.1	31.1	19.6	19.3	22.4	25.9	27.2	26.2
	2	29.7	29.1	29.9	29.5	20.9	20.9	22.2	25.5	31.8	33.7
		29.5	29.2	29.7	29.3	20.2	20.6	22.4	25.7	31.2	34.2
		29.6	29.0	30.0	29.2	20.3	20.4	22.4	25.7	32.3	> 35
D	1	31.6	29.0	33.6	32.5	26.4	25.2	25.5	29.7	> 35	30.7
		31.8	28.9	33.8	32.6	26.3	25.1	25.5	29.8	> 35	31.0
		31.8	29.0	33.5	32.3	26.5	25.3	25.5	29.4	> 35	30.8

Raw Ct values (*n* = 4 cell lines, technical replicates *n* = 3) of *ALAS1*, *RRP1*, *18S*, *PGK1* and *GUSB* in hADSC-A, -B, -C and -D under normoxic (AN1, AN2, BN1, BN2, CN1, CN2, DN1) and hypoxic (AH1, AH2, BH1, BH2, CH1, CH2, DH1) conditions. In samples where Ct values were 35 or above, gene expression was determined as being below the level of detection and these data were not included

Selection for low CV in the Hx hADSC found 8 of 78 HKG candidates screened had a $\underline{CV}$ ≤ 15%. The top 4 HKG were validated further by qRT-PCR along with *PGK1* and *18S* mRNA expression detected in four hADSC cell lines cultured under Nx and Hx conditions. Table 3 lists the raw Ct values for each HKG (*ALAS1*, *RRP1*, *18S*, *PGK1* and *GUSB)* in each hADSC (POLR2B was not detected in all hADSC but was expressed in a control cell line (keratinocytes) at raw Ct = 25.0 ± 0.92). Of the expressed HKG, GUSB showed very high Ct values in hADSC-A, -B and -D, indicating very low expression of the gene (keratinocytes showed expression at raw Ct = 25.5 ± 0.08). Hence, both POLR2B and GUSB were unsuitable HKG for hADSC in hypoxia studies due to the low to no expression levels on qRT-PCR.

**Fig. 5:**
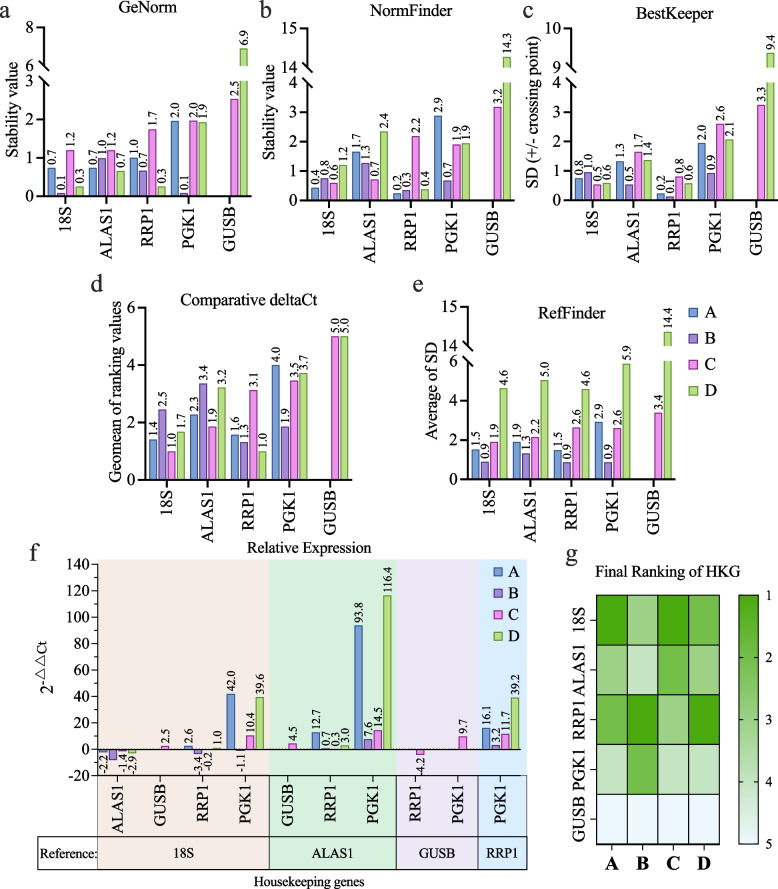
Comparative study of published methods for quantitating variance in normalised Ct values of housekeeping genes in hADSC under Nx and Hx culture conditions. Raw Ct values of the 5 HKG in hADSC under Nx and Hx conditions were normalised and ranked using **A**) GeNorm (geometric mean), **B**) NormFinder (model-based variance estimation), **C**) BestKeeper (pair-wise correlations) **D**) comparative delta-Ct, **E**) RefFinder (geometric mean of each ranking), **F**) relative expression calculated with 2^−△△Ct^ (Livak and Schmittgen 2001) using all HKG candidates as reference genes in a step-wise manner to compare between Hx and Nx, and **G**) The final ranking of all HKG candidates in each hADSC

Ct values were validated using GeNorm (geometric average), NormFinder (model-based variance estimation), BestKeeper (pair-wise correlations), comparative delta-Ct, RefFinder (geometric average of weighted ranking) and Livak (${2}^{-\Delta \Delta Ct}$) methods to quantify the stability of HKG expression in hADSC cultured under Nx and Hx conditions. Figure 5a-e summarises the ranking of the 5 HKG, where the closer each value is to 1, the more stably expressed each HKG were. Figure 5f shows the relative expression (${2}^{-\Delta \Delta Ct}$) of each gene using one of the HKG as a reference gene at each time. Based on the combined analysis of all 6 methods (Fig. 5g), *18S* and *RRP1* were identified as most stable of the 5 HKG tested in our culture conditions.

**Fig. 6:**
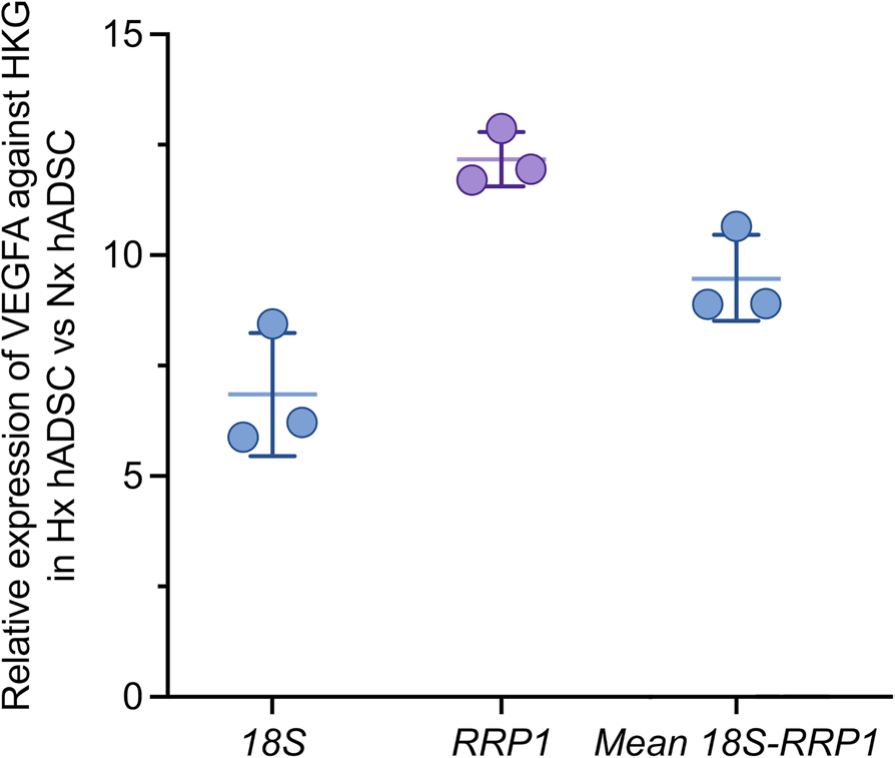
Validation of 18S and RRP1 housekeeping genes by qRT-PCR analysis for hypoxic stimulation of VEGFA expression. Relative expression of *VEGFA* in hypoxic versus normoxic treated hADSC (${2}^{-\Delta \Delta Ct}$). Relative expression was calculated using the geometric mean of both *18S* and *RRP1* reference genes (N = 3 replicates, experiment repeated twice. Data are represented as mean ± SD)

Further validation of the suitability of 18S and RRP1 as HKG (Fig. 6) was performed. The expression of a known hypoxia-regulated gene – *VEGFA*, was normalised to *18S* and *RRP1* and the levels of expression in cells treated with Hx compared to those cultured under Nx conditions. The relative expression of VEGFA was stable across 2 biological replicates of hADSC (hADSC-C and -D) cultured in hypoxia or normoxia. Figure 6 shows calculated relative expression (${2}^{-\Delta \Delta Ct}$) of *VEGFA* was up-regulated 6.8-fold ± 1.4 when using *18S* as HKG, compared to a 12-fold ± 0.6 upregulation when using *RRP1* as HKG. Used in combination, using the geometric mean of the two selected HKG, a 9.5-fold ± 1.0 upregulation in VEGFA gene expression was measured.

## Discussion

Since some typical HKG were found to be regulated in hADSC by hypoxia, this study used RNAseq datasets to screen the transcriptome for suitable HKG candidates to validate differential gene expression levels, regulated by hypoxia, by qRT-PCR. Further validation was performed with qRT-PCR and analysed using 6 established methods to calculate the variation in relative gene expression. *18S* and *RRP1* were identified as stable HKG suitable for use in hypoxia studies in hADSC in vitro. The suitability of HKG candidates was determined by having low variation in the gene expression levels across test and control samples. Furthermore, the raw Ct values of 18S and RRP1 fell within the Ct range expected of high copy number and commonly used HKG genes, which usually range between 14 to 32 raw Ct [40] (Ct values are inverse proportional to gene expression levels). In addition, the Ct of these candidate HKG were no more than 10 raw Ct values apart from the Ct of the genes-of-interest which is important to reduce variation as a result of amplification errors. qRT-PCR validation identified the following candidate HKGs suitable for hypoxia studies in hADSC, these genes have been ranked from the most stable to least stable: 1) *18S*, 2) *RRP1*, 3) *ALAS1*, 4) *PGK1* and 5) *GUSB*.

Comparison between the RNAseq-screening approach and qRT-PCR (the gold standard), identified only *RRP1* and *PGK1* as suitable HKG for hypoxia studies. The result for *GUSB*, *ALAS1* and *POLR2B* were not consistent between these methods. *18S*, an rRNA not identified in the transcriptome data since the library preparation was enriched for mRNA, was validated by qRT-PCR against *VEGFA*, a known hypoxia-regulated gene, was one of the most stable genes tested in this study, evident also in a previous study [17]. This represents a limitation in using RNAseq to identify for HKG as this approach does not account for total RNA and may exclude potential non-mRNA HKG candidates.

One criterion for HKG candidates was to be constitutively expressed in normal cell functions [1]. *18S* rRNA is a small subunit rRNA, which together with the large subunit rRNA forms ribosomes essential for mRNA translation. *RRP1,* encodes for the nuclear protein ribosomal RNA processing protein 1 homolog A, which is recruited at late stages of nucleologenesis during mitosis [41]. From the validation experiments using *VEGFA* as a known hypoxia-regulated gene, both *18S* and *RRP1* fulfilled the selection of HKG based on cellular functions, and stable replicable relative expressions with *VEGFA* in 2 biological hADSC replicates (hADSC-C and -D).

Both *18S* and *RRP1* are suitable HKG for hADSC hypoxia studies either individually, or used in combination. *18S* is expressed at a higher level (Ct ~ 20) and *RRP1* has a lower expression (Ct ~ 30), which allows users the option to select the HKG expressed at a similar level to their gene-of-interest to not overestimate relative gene expression analysis. Alternatively, according to the Minimum Information for Publication of Quantitative Real-Time PCR Experiments (MIQE) guidelines [28, 42], it is recommended to use a combination of multiple HKG using geometric mean.

Apart from stable HKG, the results also identified genes that are not suitable as HKG for hADSC hypoxia studies. *ALAS1* is a mitochondrial enzyme where cellular respiration and cell response to hypoxia takes place [43]. It was found to be significantly down-regulated (2^−△△Ct^ ≤ -1.4-fold change) when normalised to *18S* and hence not suitable HKG for hADSC hypoxia studies. *PGK1*, encoding for a glycolytic enzyme involved in glucose metabolism and a known regulator of hypoxia, was confirmed as a Hx-regulated gene in hADSC in both RNAseq and qRT-PCR results, acting as a positive control measure in gene regulation under hypoxia. *GUSB*, ranked less stable than *PGK1*, encodes for beta-glucuronidase, which is an enzyme located in the lysosomes involved in glycosaminoglycan degradation. *GUSB* was expressed at high Ct values, indicating low gene expression, suggesting a down-regulation of *GUSB* and the production of beta-glucuronidase as a response to hypoxia regulation and hence not an appropriate HKG for this study.

In addition to the genes validated in qRT-PCR, other commonly used HKG were also shown to be significantly regulated by hypoxia in hADSC (adj-P ≤ 0.05) transcriptome data. These included *GAPDH* (2^3.12^ = 8.7-fold), *HPRT* (2^0.53^ = 1.4-fold), *PGK1* (2^3.53^ = 11.6-fold), *PPIA* (2^0.78^ = 1.7-fold), *RPL13A* (2^1.23^ = 2.3-fold), *RPLP0* (2^1.58^ = 3.0-fold), *YWHAZ* (2^0.39^ = 8.7-fold) and *TFRC* (2^–3.23^ = -9.4-fold). The literature indicated that HRE were identified in the promoter regions of *PGK1* [10], *GAPDH* [13] and *TFRC* [44], but not *HPRT*, *PPIA*, *RPL13A*, *RPLP0* or *YWHAZ*, suggesting an indirect hypoxia response to these genes. Hence, this study provides added information to exclude genes that were unsuitable as HKG for Hx hADSC, in the absence of HRE in corresponding promoter regions.

In comparison to the only other study in the literature comparing HKG in hADSC cultured under hypoxic conditions [20], this study identified different findings for *18S* and *GUSB* being suitable HKG, but also similar findings in identifying unsuitable HKG such as *GAPDH*. While it is accepted that *GAPDH* is regulated by hypoxia and not suitable to be used as a HKG, the difference in findings for *18S* and *GUSB* might be contributed to hADSC being cultured in slightly different growth conditions in Fink et al., such as high versus low passages, and hypoxia cultures over 1 and 2 weeks. Our culture conditions have been optimised for the experimental question, and hence reinforcing the need to validate suitable HKG according to experimental design.

### Limitations of study

RNA sequencing was performed in two separate batches and the differential expression analysis did not correct for batch effects. However, the selection of HKG using RNAseq data for CV calculation was independently performed between the hADSC lines. As the second sequencing experiment (for hADSC-A, -B, -C and -D) was sequenced more in-depth (25 to 57 million reads) than the first (for hADSC-E at 5.9 to 8.7 million reads), more confidence was given if CV ≤ 0.15 was more consistent in hADSC-A, -B, -C and -D when selecting for HKG after the calculations.

This study described a straightforward RNAseq-based screening approach to identify potential HKG, with the assumption that normalised count reads represent gene expressions. However, the TPM normalisation method may underestimate genes that are expressed at very low levels. For example, hADSC-C mean expression for *CDKN1A* was 163.5 TPM ± 19.1 TPM at CV 11.7%, but for *CHFR* was 0.6 ± 0.1 TPM at CV = 23.1%. In this scenario, *CDKN1A* would be defined as “stable expression” and included for subsequent analysis, but *CHFR*, where there was only 0.1 TPM standard deviation of count reads, would be excluded. This may be resolved if the selection for potential HKG is performed for genes expressed at similar levels as specific gene-of-interests of the study.

Additionally, this study may be limited by specificity of probe/primer designs in qRT-PCR, as *POLR2B* was expressed in both RNAseq normalised counts, but when used for qRT-PCR validation, were found to not be expressed at detectable levels. Further evaluation of qRT-PCR primers for *POLR2B* isoforms in hADSC may be warranted in future studies.

## Conclusions

In conclusion, this study found RNAseq data able to identify candidate housekeeping genes for use in hypoxia studies of human adipose-derived mesenchymal stem cells. After validation by qRT-PCR, *18S* rRNA and *RRP1* were identified as suitable HKG candidates and are recommended as stable housekeeping genes for future hypoxia-induced gene expression studies in hADSC. The differential expression of several commonly used housekeeping genes, provided further evidence to eliminate them as suitable for HKG in future hypoxia studies of human adipose-derived stem cells.

## Materials and methods

### Cell culture maintenance

hADSC from 5 donors (A, B, C, D and E) were purchased from Lonza (Lonza, Walkersville, Maryland) or ZenBio (ZenBio, Research Triangle Park, North Carolina), where cells were previously characterised (general information on hADSC donors and cell characterisation are provided in Additional file 5). hADSC were cultured in Dulbecco’s Modified Eagle’s medium (DMEM) containing 1 g/L D-glucose (Gibco, Carlsbad, California), 2 mM GlutaMAX™ (hADSC-E in 4 mM L-glutamine) and 110 mg/L Sodium Pyruvate, supplemented with 10% foetal bovine serum (FBS, Bovogen, Keilor East, Australia) and 1% Penicillin–Streptomycin (P/S; Gibco, Carlsbad, California). Spent media were replaced every 2–3 days. Cells were incubated in standard culture conditions of 37 °C, with 5% carbon dioxide (CO_2_) and 21% atmospheric oxygen (O_2_) in a humidified cell culture incubator unless otherwise specified, and passaged at approximately 80% confluence. hADSC were used between passage 5 to 8.

A human eardrum keratinocyte cell line characterised previously [45] was cultured in DMEM containing 4.5 g/L D-glucose (Gibco, Carlsbad, California), 4 mM L-glutamine) and 110 mg/L Sodium Pyruvate, supplemented with 10% FBS and 1% P/S. Spent media were replaced every 3–4 days. Cells were incubated in standard culture conditions of 37 °C, with 5% CO_2_ and 21% atmospheric oxygen (O_2_) in a humidified cell culture incubator and passaged at approximately 90% confluence. Keratinocytes of passage 36 were used.

### Hypoxia conditioning

hADSC were cultured to 80–90% confluence in 75 cm^2^ tissue culture flasks, rinsed with phosphate-buffered saline (PBS), pH 7.2 (Gibco, Carlsbad, California), and cultured in serum-free DMEM for at least 8 h before replacing with fresh serum-free medium for a further 48 h. hADSC at this step were either incubated in standard (Nx; 37 °C, 5% CO_2_) or Hx conditions in the GENbox Jar (BioMérieux, Craponne, France) produced by an anaerobic atmosphere generator sachet (BioMérieux, Craponne, France) at 37 °C. A hypoxia indicator strip (BioMérieux, Craponne, France) was included in the sealed chamber to confirm gas composition (< 0.1% O_2_ and 15% CO_2_). At the end of the 48-h incubation, total RNA was extracted.

### RNA extraction

Total RNA from hADSC-E was extracted using the FavorPrep™ Tissue total RNA Mini Kit (Favorgen Biotech Corp, Ping Tung, Taiwan) according to the manufacturer’s protocol, before eluted RNA was cleaned up of genomic DNA (gDNA) using the RNeasy Plus Micro kit (Qiagen, Hilden, Germany). Total RNA from hADSC-A, -B, -C, -D, and keratinocytes were extracted using the RNeasy Plus Micro kit according to the manufacturer’s instructions. Briefly, cells were lysed and gDNA was removed via the gDNA Eliminator spin column. Total RNA was then bound, collected and washed on the RNeasy MinElute spin column before being eluted at approximately 14 μL per sample. All RNA samples were stored at -80 °C until required for RNA sequencing and cDNA synthesis.

RNA samples were submitted to the Australia Genome Research Facility (AGRF, Melbourne, Australia) for quality control, library preparation and RNA sequencing. Briefly, RNA samples were quantified using a 2100 Bioanalyzer (Agilent Technologies, Santa Clara, California) where samples of at least an RNA Integrity Number (RIN) of ≥ 8.0 and an A260/280 ratio of 1.8 – 2.0 were accepted. RNA quantification results are provided in Additional file 6.

### Library preparation and RNA sequencing

For all samples, library preparation was performed using the Illumina TruSeq® Stranded mRNA kit according to the manufacturer’s protocol (Illumina, San Diego, California). Briefly, mRNA was purified and fragmented for double-stranded cDNA synthesized using specific methods to improve strand specificity. Adapters were ligated to DNA fragments to prepare for hybridization onto a flow cell during RNA sequencing, and enriched by amplification.

For hADSC-E, samples were processed earlier in a separate batch to the rest. RNA sequencing was performed on the Illumina HiSeq 2500 platform High Output Mode using the HiSeq Control Software (HCS) v2.2.68 and Real Time Analysis (RTA) v1.18.66.3 running on the Illumina sequencing computer for single-end reads at 50 bp. The Illumina bcl2fastq 2.18.0.12 pipeline was used next to generate the sequence data.

For hADSC-A, -B, -C and -D, RNA sequencing was performed on the Illumina NovaSeq 2500 platform High Output Mode using the NovaSeq Control Software (NCS) v 1.6.0 and Real Time Analysis (RTA) v 3.4.4 performing real-time based calling on the NovaSeq instrument computer for single-end reads at 100 bp. The Illumina bcl2fastq v 2.20.0.422 pipeline was used next to generate the sequence data. All RNAseq files were uploaded onto ArrayExpress accession E-MTAB-9979.

### Bioinformatic analysis

RNAseq raw fastq files were checked using the FastQC (v 0.11.9) tool [46] to evaluate the quality of the sequenced samples. Reads were trimmed with Trimmomatic (v 0.36) [47] with parameters recommended in the Trimmomatic tool manual for single-end reads. Trimmed reads were then aligned to the GrCh38 human reference genome assembly obtained from Ensembl using the HISAT2 (v 2.1.0) tool [48], with the default parameters in the HISAT2 tool manual. Mapped reads were processed using SamBamba (v 0.6.6) [49]. For hADSC-A, -B, -C and -D, lane 1 files were merged with their corresponding repeated lane 2 files. Multimappers were filtered out using a mapping quality threshold of ≥ 5. Sorted and filtered Bam files were used as input for the featureCounts function in subread [50] to count each transcript to the GrCh38.90 reference genome. Sequencing details and processing steps are attached in Additional file 7. Count files (number of reads per transcript counted in data files) were parsed to include the corresponding Ensembl Gene IDs, before analysing with DESeq2 (v 1.26.0) [51] via R studio.

Differential expression analyses were performed using DESeq2 to compare differential gene expressions from hADSC cultured in Hx against Nx. Low count reads (< 10) were removed and DESeq2 was used with the multi-factor design for “conditions” (Hx vs Nx) and “cell types” (hADSC-A, -B, -C, -D and -E). Count data were transformed using regularised logarithmic transformation (rlog function) in DESeq2 to compare between datasets in the clustering analyses. Differential expression analysis was performed on raw count data and results were shrunk using the Apeglm function [52] for better estimation of distribution and exported as L2FC. Transcripts were considered differentially expressed (DE) if L2FC ≥ 2 at adjusted-P ≤ 0.05.

In addition to the L2FC data, raw count reads from all 5 hADSC were also normalised to transcripts per million (TPM) by normalising for both gene lengths and sequence depths.

All analysis performed in R for this study can be found in Additional file 4.

### Ranking method

From the literature, a list of 78 previously known HKG were curated. L2FC and DESeq2-normalised values were extracted from the differential expression analysis data of the 5 hADSC, and TPM were calculated using the formula below, for the 78 HKG.

$$Transcripts\;per\;million\;\left(TPM\right)=Raw\;counts\div\frac{Gene\;length}{1000}\div Sequencing\;depth$$

The CV of each HKG between Hx and Nx was calculated with TPM or DE data using the following formula below, for all 5 hADSC (CV_ABCDE_) as well as individually (CV_A_, CV_B_, CV_C_, CV_D_, CV_E_):

$$Co\;efficient\;of\;Variance\;\left(CV\right)=\frac{{Standard\;Deviation}_{Hx\;and\;Nx(TPMorDE)}}{M{ean}_{Hx\;and\;Nx\;(TPMorDE)}}$$

An average of CV ($\underline{CV}$) was first calculated using only CV ≤ 0.15. For example, *ALAS1* had 7 CV values ≤ 0.15, the average CV was calculated by averaging the 7 CV values only. Next, the top 8 genes were selected to normalise for both CV and L2FC by dividing each value with the smallest corresponding values for an arbitrary score. For example, from the averaged CV, *ALAS1* was the smallest at 0.013 so average CV of all other genes were normalised to 0.013, and of the L2FC, *GUSB* was the smallest (closest to 0) at 0.01 so L2FC of all other genes were normalised to 0.01. These two arbitrary scores were then summed and ranked from the smallest to the largest where the top 4 HKG were selected for validation via qRT-PCR. Additional file 3 contains all 78 HKG data, calculations, and ranking.

### cDNA synthesis

Complementary DNA was synthesised using the RT^2^ Omniscript kit (Qiagen, Hilden, Germany) according to manufacturer’s instructions. Briefly, 1 μg of template RNA was reverse transcribed in accordance with the manufacturer’s protocol for each sample, together with the corresponding no reverse transcriptase controls (NRT) and no template controls (NTC). For each reaction, 1 μM oligo(dT)_15_ primers (Promega Corporation, Fitchburg, Wisconsin) were used, where reactions were incubated at 37 °C for 60 min.

### qRT-PCR

qRT-PCR was performed using the QuantiFast Probe PCR kit (Qiagen, Hilden, Germany) on cDNA generated from hADSC-A, -B, -C and -D RNA samples. Gene expressions were performed using FAM-labelled and VIC-labelled TaqMan Assay probes (Thermo Fisher Scientific, Waltham, Massachusetts) as listed below in Table 4. *18S* was not included in our RNAseq since it’s an rRNA, *VEGFA* was to validate the known regulation by hypoxia. Briefly, 2.5 μL of cDNA, 5 μL of 2 × PCR Mastermix, 0.5 μL of TaqMan Assay probes and RNase-free H_2_O were combined to a total of 10 μL per reaction per well in a white 96-well full-skirt plate, along with appropriate controls for NRT and NTC. qRT-PCR was performed on the CFX Real-Time PCR Detection System (Bio-Rad Laboratories, Hercules, California) under the following conditions: 95 °C for 3 min, and 40 cycles of denaturation at 95 °C for 3 s and annealing at 60 °C for 30 s. Threshold cycles (Ct) were determined using the exponential growth phase and the baseline signal from fluorescence versus cycle number plots.

**Table 4:** Description of TaqMan Assay probes used in qRT-PCR validation of housekeeping genes

Gene	Label	Amplicon (bp)	Assay Design	Catalogue No	Reference
*ALAS1*	FAM	77	Spanning Exon 9—10	Hs00167441_m1	NM_000688.5
*GUSB*	FAM	96	Spanning Exon 8—9	Hs00939627_m1	NM_000181.3
*POLR2B*	FAM	58	Spanning Exon 6—7	Hs00946293_m1	NM_000938.2
*RRP1*	FAM	75	Spanning Exon 4—5	Hs01002953_m1	NM_003683.5
*VEGFA*	FAM	59	Spanning Exon 3—4	Hs00900055_m1	NM_001025366.2
*18S*	VIC	187	Within single exon	Hs99999901_s1	X03205.1
*PGK1*	VIC	75	Spanning Exon 4—5	Hs99999906_m1	NM_000291.3

Information of TaqMan Assay probes used, and the NCBI Reference Sequences for the corresponding genes. FAM: 5(6)-carboxy-fluorescein; VIC: 2’-chloro-7’phenyl-1,4-dichloro-6-carboxy-fluorescein

### Validation of HKG stability

Using RefFinder [35], raw Ct values for each HKG of all 4 hADSC lines were entered according to the instructions to calculate for geometric average (GeNorm), model-based variance estimation (NormFinder), pair-wise correlations (BestKeeper), $\Delta Ct$ method, as well as the geometric mean of the weighted ranks from the 4 methods to be expressed as RefFinder rank. Then, relative gene expression levels were determined using ${2}^{-\Delta \Delta Ct}$ method [39] for each hADSC to compare Hx (test) against Nx (control). The overall rank was averaged for each HKG. Relative gene expression levels (*n* = 3) using ${2}^{-\Delta \Delta Ct}$ was also performed for *VEGFA* against *18S* and *RRP1* in 2 biological replicates of hADSC in Hx (test) against Nx (control).

## Supplementary Information

**Additional file 1.** List of 114 hypoxia-inducible genes, gene symbol, and gene ID.

**Additional file 2.** Preliminary qRT-PCR data analysis of RPL13A, TBP, GAPDH, B2M, VEGFA, using RNA extracted of hADSC cultured in normal and hypoxic conditions.

**Additional file 3.** Ranking of normalised counts of RNAseq data of Nx-hADSC and Hx-hADSC curated for selected HKG candidates and calculated for CV.

**Additional file 4.** R markdown script of differential expression analysis of RNAseq data using DESeq2.

**Additional file 5.** Summary of hADSC donor information and cell characterisation used in this study.

**Additional file 6.** Quality control and qualification results of RNA extracted from hADSC.

**Additional file 7.** RNAseq processing efficiencies represented as percentage counts of original sequenced reads

## Data Availability

The datasets generated during this study are available in the ArrayExpress repository with the accession code E-MTAB-9979. For correspondence and material requests, please address them to HTO.

## Abbreviations

qRT-PCR: Real-time quantitative polymerase chain reaction
cDNA: Complementary DNA
POLR2B: RNA polymerase II subunit B
ALAS1: 5’-Aminolevulinate synthase 1
GUSB: Glucuronidase beta
RRP1: Ribosomal RNA processing 1
18S: 18S Ribosomal RNA
PGK1: Phosphoglycerate Kinase 1
VEGFA: Vascular endothelial growth factor A
Ct: Threshold cycles
NRT: No reverse transcriptase control
NTC: No template control
HKG: Housekeeping gene
TPM: Transcript per million
Hx: Hypoxic
Nx: Normoxic
L2FC: Log 2 Fold Change
CV: Coefficient of Variance
hADSC: Human adipose-derived stem cells
ACTB: Beta-actin
RPLP0: Ribosomal protein lateral stalk subunit P0
TFRC: Transferrin receptor
B2M: Beta-2-microglobulin
RPL13A: Ribosomal protein L13a
RPII: RNA polymerase II
28S: 28S rRNA
HPRT1: Hypoxanthine phosphoribosyltransferase 1
YWHAZ: Tyrosine 3/tryptophan 5-monooxygenase activation protein
TBP: TATA-box binding protein
GAPDH: Glyceraldehyde 3-phosphate dehydrogenase
PPIA: Peptidylprolyl isomerase A
mRNA: Messenger RNA
HRE: Hypoxia response elements
HIF-1: Hypoxia-inducible factor -1
