# Supplementary material for: Using RNA-seq to identify suitable housekeeping genes for hypoxia studies in human adipose-derived stem cells
Source: BMC Mol Cell Biol. 2023 Apr 17;24:16. doi: 10.1186/s12860-023-00475-4 (PMC10108514; doi:10.1186/s12860-023-00475-4)
Supplement: Supplementary file 1 — Additional file 1. List of 114 hypoxia-inducible genes, gene symbol, and gene ID. [file 12860_2023_475_MOESM1_ESM.pdf]

Additional File 1. List of 114 hypoxia-inducible genes, gene symbol, and gene ID

Hypoxia.inducible.gene	Approved.name	GeneID
ABCG2	ATP binding cassette subfamily G member 2 (Junior blood group)	ENSG00000118777
ADM	adrenomedullin	ENSG00000148926
ALDOA	aldolase, fructose-bisphosphate A	ENSG00000149925
ALDOC	aldolase, fructose-bisphosphate C	ENSG00000109107
ANGPT1	angiopoietin 1	ENSG00000154188
ANGPT2	angiopoietin 2	ENSG00000091879
ANGPTL4	angiopoietin like 4	ENSG00000167772
ANKRD37	ankyrin repeat domain 37	ENSG00000186352
AURKA	aurora kinase A	ENSG00000087586
BHLHE41	basic helix-loop-helix family member e41	ENSG00000123095
BNIP3	BCL2 interacting protein 3	ENSG00000176171
BNIP3L	BCL2 interacting protein 3 like	ENSG00000104765
CA9	carbonic anhydrase 9	ENSG00000107159
CTGF	cellular communication network factor 2	ENSG00000118523
CITED2	Cbp/p300 interacting transactivator with Glu/Asp rich carboxy-terminal domain 2	ENSG00000164442
COX4I2	cytochrome c oxidase subunit 4I2	ENSG00000131055
CP	ceruloplasmin	ENSG00000047457
CTSC	cathepsin C	ENSG00000109861
CXCL12	C-X-C motif chemokine ligand 12	ENSG00000107562
CXCR4	C-X-C motif chemokine receptor 4	ENSG00000121966
CYBB	cytochrome b-245 beta chain	ENSG00000165168
DDIT4	DNA damage inducible transcript 4	ENSG00000168209
DEC1	deleted in esophageal cancer 1	ENSG00000173077
EDN1	endothelin 1	ENSG00000078401
EGLN1	egl-9 family hypoxia inducible factor 1	ENSG00000135766
EGLN3	egl-9 family hypoxia inducible factor 3	ENSG00000129521
ENG	endoglin	ENSG00000106991
ENO1	enolase 1	ENSG00000074800
EPX	eosinophil peroxidase	ENSG00000121053
FECH	ferrochelatase	ENSG00000066926
FLT1	fms related receptor tyrosine kinase 1	ENSG00000102755
FURIN	furin, paired basic amino acid cleaving enzyme	ENSG00000140564
GADD45A	growth arrest and DNA damage inducible alpha	ENSG00000116717
GAPDH	glyceraldehyde-3-phosphate dehydrogenase	ENSG00000111640
GPI	glucose-6-phosphate isomerase	ENSG00000105220
GPX3	glutathione peroxidase 3	ENSG00000211445
HK1	hexokinase 1	ENSG00000156515
HK2	hexokinase 2	ENSG00000159399
HMOX1	heme oxygenase 1	ENSG00000100292
HSP90B1	heat shock protein 90 beta family member 1	ENSG00000166598
ID2	inhibitor of DNA binding 2	ENSG00000115738
IGF2	insulin like growth factor 2	ENSG00000167244
IGFBP1	insulin like growth factor binding protein 1	ENSG00000146678
IGFBP3	insulin like growth factor binding protein 3	ENSG00000146674
IGHD6-13	immunoglobulin heavy diversity 6-13	ENSG00000211920
ITGB2	integrin subunit beta 2	ENSG00000160255
KDR	kinase insert domain receptor	ENSG00000128052
L1CAM	L1 cell adhesion molecule	ENSG00000198910
LDHA	lactate dehydrogenase A	ENSG00000134333
LEP	leptin	ENSG00000174697
LGALS1	galectin 1	ENSG00000100097
LONP1	lon peptidase 1, mitochondrial	ENSG00000196365
LOX	lysyl oxidase	ENSG00000113083
LOXL2	lysyl oxidase like 2	ENSG00000134013
LOXL4	lysyl oxidase like 4	ENSG00000138131
MCL1	MCL1 apoptosis regulator, BCL2 family member	ENSG00000143384
ABCB1	ATP Binding Cassette Subfamily B Member 1	ENSG00000085563
MET	MET proto-oncogene, receptor tyrosine kinase	ENSG00000105976
MMP1	matrix metallopeptidase 1	ENSG00000196611
MMP14	matrix metallopeptidase 14	ENSG00000157227
MMP2	matrix metallopeptidase 2	ENSG00000087245
MMP9	matrix metallopeptidase 9	ENSG00000100985

MXI1	MAX interactor 1, dimerization protein	ENSG00000119950
NDRG1	N-myc downstream regulated 1	ENSG00000104419
NOS2	nitric oxide synthase 2	ENSG00000007171
NOS3	nitric oxide synthase 3	ENSG00000164867
NPM1	nucleophosmin 1	ENSG00000181163
NPPA	natriuretic peptide A	ENSG00000175206
NR4A1	nuclear receptor subfamily 4 group A member 1	ENSG00000123358
NT5E	5'-nucleotidase ecto	ENSG00000135318
P4HA1	prolyl 4-hydroxylase subunit alpha 1	ENSG00000122884
PCK2	phosphoenolpyruvate carboxykinase 2, mitochondrial	ENSG00000100889
PDGFB	platelet derived growth factor subunit B	ENSG00000100311
PDK1	pyruvate dehydrogenase kinase 1	ENSG00000152256
PFKFB1	6-phosphofructo-2-kinase/fructose-2,6-biphosphatase 1	ENSG00000158571
PFKFB2	6-phosphofructo-2-kinase/fructose-2,6-biphosphatase 2	ENSG00000123836
PFKFB3	6-phosphofructo-2-kinase/fructose-2,6-biphosphatase 3	ENSG00000170525
PFKFB4	6-phosphofructo-2-kinase/fructose-2,6-biphosphatase 4	ENSG00000114268
PFKL	phosphofructokinase, liver type	ENSG00000141959
PGF	placental growth factor	ENSG00000119630
PGK1	phosphoglycerate kinase 1	ENSG00000102144
PGM1	phosphoglucomutase 1	ENSG00000079739
PKM	pyruvate kinase M1/2	ENSG00000067225
PLAUR	plasminogen activator, urokinase receptor	ENSG00000011422
PMAIP1	phorbol-12-myristate-13-acetate-induced protein 1	ENSG00000141682
POU5F1	POU class 5 homeobox 1	ENSG00000204531
PPP5C	protein phosphatase 5 catalytic subunit	ENSG00000011485
RORA	RAR related orphan receptor A	ENSG00000069667
SERPINE1	serpin family E member 1	ENSG00000106366
SLC16A3	solute carrier family 16 member 3	ENSG00000141526
SLC2A1	solute carrier family 2 member 1	ENSG00000117394
SLC2A3	solute carrier family 2 member 3	ENSG00000059804
SLC9A1	solute carrier family 9 member A1	ENSG00000090020
SNAI1	snail family transcriptional repressor 1	ENSG00000124216
SNAI2	snail family transcriptional repressor 2	ENSG00000019549
SOD2	superoxide dismutase 2	ENSG00000112096
STC2	stanniocalcin 2	ENSG00000113739
TCF3	transcription factor 3	ENSG00000071564
TERT	telomerase reverse transcriptase	ENSG00000164362
TF	transferrin	ENSG00000091513
TFF3	trefoil factor 3	ENSG00000160180
TFRC	transferrin receptor	ENSG00000072274
TGFA	transforming growth factor alpha	ENSG00000163235
TGFB3	transforming growth factor beta 3	ENSG00000119699
TIMP1	TIMP metallopeptidase inhibitor 1	ENSG00000102265
TKT	transketolase	ENSG00000163931
TKTL2	transketolase like 2	ENSG00000151005
TPI1	triosephosphate isomerase 1	ENSG00000111669
TWIST1	twist family bHLH transcription factor 1	ENSG00000122691
VEGFA	vascular endothelial growth factor A	ENSG00000112715
VIM	vimentin	ENSG00000026025
WT1	WT1 transcription factor	ENSG00000184937
ZEB1	zinc finger E-box binding homeobox 1	ENSG00000148516
ZEB2	zinc finger E-box binding homeobox 2	ENSG00000169554
