# Supplementary material for: Using RNA-seq to identify suitable housekeeping genes for hypoxia studies in human adipose-derived stem cells
Source: BMC Mol Cell Biol. 2023 Apr 17;24:16. doi: 10.1186/s12860-023-00475-4 (PMC10108514; doi:10.1186/s12860-023-00475-4)
Supplement: Supplementary file 4 — Additional file 4. R markdown script of differential expression analysis of RNAseq data using DESeq2. [file 12860_2023_475_MOESM4_ESM.pdf]

Additional File 4. R markdown script of
Differential Expression Analysis of RNAseq data using DESeq2
07/07/2021

Contents

Differential Expression Analysis **1**
- Input data . . . 1
- Run DESeq2 . . . 3
- Data Visualization using Log fold change shrinkage . . . 4
- Plot volcano plot . . . 6
- Using ggplot2 (+ ggrepel), plot L2FC . . . 6

Data quality assessment - sample clustering and visualization **7**
- Heatmap of hADSC Hx vs Nx - Transcript variant . . . 7
- Heatmap of hADSC Hx vs Nx - sample-to-sample distance . . . 8
- PCA plot of samples . . . 8

Selecting for Housekeeping genes **9**

Differential Expression Analysis

Input data

Merge raw counts for all 5 samples

Assign specific columns to create "cts" dataframe

```
write.table(hADSCABCDE_HxNx, file = "hADSCABCDE_HxNx_cts.txt", quote = F, sep = "\t",
            na = "NA", row.names = F, col.names = T)
cts <- hADSCABCDE_HxNx[,c("Geneid", "AH1", "AH2", "AH3", "AN1", "AN2", "AN3",
                          "BH1", "BH2", "BH3", "BN1", "BN2", "BN3",
                          "CH1", "CH2", "CH3", "CN1", "CN2", "CN3",
                          "DH1", "DH2", "DH3", "DN1", "DN2", "DN3",
                          "EH1", "EH2", "EN1", "EN2")]
```

Set up column data information to be "coldata"

```r
cts <- as.matrix(read.csv(file = 'hADSCABCDE_HxNx_cts.txt', sep="\t",
                          row.names="Geneid"))
conditions <- c("Hx", "Hx", "Hx", "Nx", "Nx", "Nx", "Hx", "Hx", "Hx",
                "Nx", "Nx", "Nx", "Hx", "Hx", "Hx", "Nx", "Nx", "Nx",
                "Hx", "Hx", "Hx", "Nx", "Nx", "Nx", "Hx", "Hx", "Nx", "Nx")
celltypes <- c("hADSCA", "hADSCA", "hADSCA", "hADSCA", "hADSCA", "hADSCA",
               "hADSCB", "hADSCB", "hADSCB", "hADSCB", "hADSCB", "hADSCB",
               "hADSCC", "hADSCC", "hADSCC", "hADSCC", "hADSCC", "hADSCC",
               "hADSCD", "hADSCD", "hADSCD", "hADSCD", "hADSCD", "hADSCD",
               "hADSCE", "hADSCE", "hADSCE", "hADSCE")
sampleA1 <- colnames(hADSCABCDE_HxNx)[which(names(hADSCABCDE_HxNx) == "AH1")]
sampleA2 <- colnames(hADSCABCDE_HxNx)[which(names(hADSCABCDE_HxNx) == "AH2")]
sampleA3 <- colnames(hADSCABCDE_HxNx)[which(names(hADSCABCDE_HxNx) == "AH3")]
sampleA4 <- colnames(hADSCABCDE_HxNx)[which(names(hADSCABCDE_HxNx) == "AN1")]
sampleA5 <- colnames(hADSCABCDE_HxNx)[which(names(hADSCABCDE_HxNx) == "AN2")]
sampleA6 <- colnames(hADSCABCDE_HxNx)[which(names(hADSCABCDE_HxNx) == "AN3")]
sampleB1 <- colnames(hADSCABCDE_HxNx)[which(names(hADSCABCDE_HxNx) == "BH1")]
sampleB2 <- colnames(hADSCABCDE_HxNx)[which(names(hADSCABCDE_HxNx) == "BH2")]
sampleB3 <- colnames(hADSCABCDE_HxNx)[which(names(hADSCABCDE_HxNx) == "BH3")]
sampleB4 <- colnames(hADSCABCDE_HxNx)[which(names(hADSCABCDE_HxNx) == "BN1")]
sampleB5 <- colnames(hADSCABCDE_HxNx)[which(names(hADSCABCDE_HxNx) == "BN2")]
sampleB6 <- colnames(hADSCABCDE_HxNx)[which(names(hADSCABCDE_HxNx) == "BN3")]
sampleC1 <- colnames(hADSCABCDE_HxNx)[which(names(hADSCABCDE_HxNx) == "CH1")]
sampleC2 <- colnames(hADSCABCDE_HxNx)[which(names(hADSCABCDE_HxNx) == "CH2")]
sampleC3 <- colnames(hADSCABCDE_HxNx)[which(names(hADSCABCDE_HxNx) == "CH3")]
sampleC4 <- colnames(hADSCABCDE_HxNx)[which(names(hADSCABCDE_HxNx) == "CN1")]
sampleC5 <- colnames(hADSCABCDE_HxNx)[which(names(hADSCABCDE_HxNx) == "CN2")]
sampleC6 <- colnames(hADSCABCDE_HxNx)[which(names(hADSCABCDE_HxNx) == "CN3")]
sampleD1 <- colnames(hADSCABCDE_HxNx)[which(names(hADSCABCDE_HxNx) == "DH1")]
sampleD2 <- colnames(hADSCABCDE_HxNx)[which(names(hADSCABCDE_HxNx) == "DH2")]
sampleD3 <- colnames(hADSCABCDE_HxNx)[which(names(hADSCABCDE_HxNx) == "DH3")]
sampleD4 <- colnames(hADSCABCDE_HxNx)[which(names(hADSCABCDE_HxNx) == "DN1")]
sampleD5 <- colnames(hADSCABCDE_HxNx)[which(names(hADSCABCDE_HxNx) == "DN2")]
sampleD6 <- colnames(hADSCABCDE_HxNx)[which(names(hADSCABCDE_HxNx) == "DN3")]
sampleE1 <- colnames(hADSCABCDE_HxNx)[which(names(hADSCABCDE_HxNx) == "EH1")]
sampleE2 <- colnames(hADSCABCDE_HxNx)[which(names(hADSCABCDE_HxNx) == "EH2")]
sampleE3 <- colnames(hADSCABCDE_HxNx)[which(names(hADSCABCDE_HxNx) == "EN1")]
sampleE4 <- colnames(hADSCABCDE_HxNx)[which(names(hADSCABCDE_HxNx) == "EN2")]
samples <- c(sampleA1, sampleA2, sampleA3, sampleA4, sampleA5, sampleA6,
             sampleB1, sampleB2, sampleB3, sampleB4, sampleB5, sampleB6,
             sampleC1, sampleC2, sampleC3, sampleC4, sampleC5, sampleC6,
             sampleD1, sampleD2, sampleD3, sampleD4, sampleD5, sampleD6,
             sampleE1, sampleE2, sampleE3, sampleE4)
coldata <- data.frame(conditions = conditions, celltypes = celltypes,
                      row.names = samples)
all(rownames(coldata) %in% colnames(cts))
```

```
## [1] TRUE
```

```r
cts <- cts[,rownames(coldata)]
all(rownames(coldata) == colnames(cts))
```

```
## [1] TRUE
```

Run DESeq2

Load packages

Upload dataframe from matrix into DESeq and assign it "dds"

```
dds <- DESeqDataSetFromMatrix(cts,
                              coldata,
                              ~ conditions + celltypes)
dds
```

```
## class: DESeqDataSet
## dim: 58243 28
## metadata(1): version
## assays(1): counts
## rownames(58243): ENSG00000000003 ENSG00000000005 ... ENSG00000284747
##   ENSG00000284748
## rowData names(0):
## colnames(28): AH1 AH2 ... EN1 EN2
## colData names(2): conditions celltypes
```

Filtered for at least 10 raw counts

```
keep <- rowSums(counts(dds)) >= 10
dds <- dds[keep,]
```

Differential Analysis function on data

```
dds <- DESeq(dds)
```

```
## estimating size factors

## estimating dispersions

## gene-wise dispersion estimates

## mean-dispersion relationship

## final dispersion estimates

## fitting model and testing
```

```
dds
```

```
## class: DESeqDataSet
## dim: 24534 28
## metadata(1): version
## assays(4): counts mu H cooks
## rownames(24534): ENSG00000000003 ENSG00000000419 ... ENSG00000284740
##   ENSG00000284747
## rowData names(38): baseMean baseVar ... deviance maxCooks
## colnames(28): AH1 AH2 ... EN1 EN2
## colData names(3): conditions celltypes sizeFactor
```

Change cutoff value for adjusted p value (padj)

```
res <- results(dds, name="conditions_Hx_vs_Nx")
resultsNames(dds)
```

```
## [1] "Intercept"                  "conditions_Hx_vs_Nx"
## [3] "celltypes_hADSCB_vs_hADSCA" "celltypes_hADSCC_vs_hADSCA"
## [5] "celltypes_hADSCD_vs_hADSCA" "celltypes_hADSCE_vs_hADSCA"
```

```
res <- DESeq2::results(dds, contrast=list("conditions_Hx_vs_Nx"), cooksCutoff = 0.99,
                       independentFiltering = T, alpha = 0.05, pAdjustMethod = "BH")
sum(res$padj < 0.05, na.rm=T)
```

```
## [1] 14179
```

```
summary(res)
```

```
##
## out of 24534 with nonzero total read count
## adjusted p-value < 0.05
## LFC > 0 (up)       : 7166, 29%
## LFC < 0 (down)     : 7013, 29%
## outliers [1]       : 425, 1.7%
## low counts [2]     : 1895, 7.7%
## (mean count < 1)
## [1] see 'cooksCutoff' argument of ?results
## [2] see 'independentFiltering' argument of ?results
```

Data Visualization using Log fold change shrinkage

```
hist(res$pvalue, breaks=50, col="grey")
```

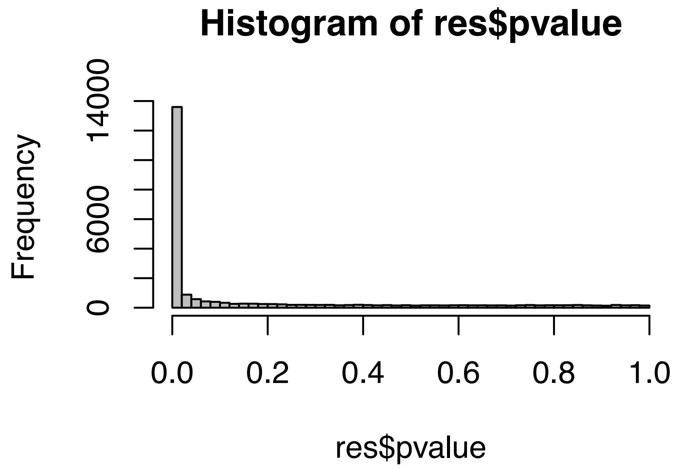

Use “apeglm” setting

```
resLFC <- lfcShrink(dds=dds, coef=2, type="apeglm")
```

```
## using 'apeglm' for LFC shrinkage. If used in published research, please cite:
##     Zhu, A., Ibrahim, J.G., Love, M.I. (2018) Heavy-tailed prior distributions for
##     sequence count data: removing the noise and preserving large differences.
##     Bioinformatics. https://doi.org/10.1093/bioinformatics/bty895
```

```
plotMA(res, ylim=c(-15,15))
abline(h=c(-1,1), col="dodgerblue", lwd=2)
abline(h=c(-2,2), col="green4", lwd=2)
plotMA(resLFC, ylim=c(-20,20))
abline(h=c(-1,1), col="dodgerblue", lwd=2)
abline(h=c(-2,2), col="green4", lwd=2)
```

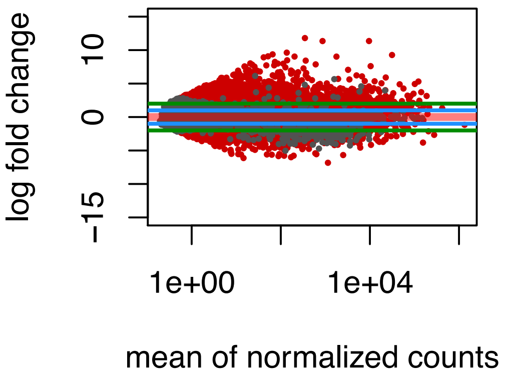

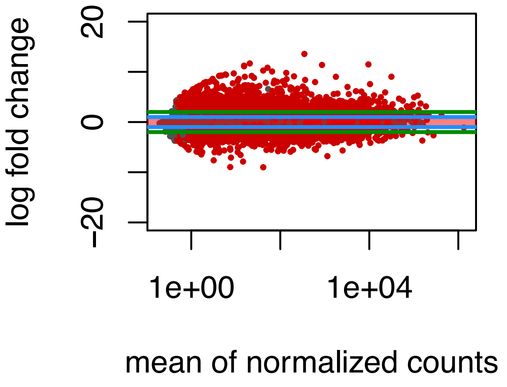

Merge results and export

```
resdata <- merge(as.data.frame(res), as.data.frame(counts(dds, normalized=T)),
                 by="row.names", sort=F)
names(resdata)[1] <- "GeneID"
resNormdata <- merge(as.data.frame(resLFC), as.data.frame(counts(dds, normalized=T)),
                     by="row.names", sort=F)
names(resNormdata)[1] <- "GeneID"
write.csv(resdata, file="hADSCABCDE_HxNx_DESeq2-originalresults.csv")
write.csv(resNormdata, file="hADSCABCDE_HxNx_DESeq2-LFCshrunkresults.csv")
GeneID_Symbol = read.delim(file = 'Gene_id_gene_names.txt', header = T, sep = "")
resdata_Sym <- merge(resdata, GeneID_Symbol, by.x = "GeneID",
                     by.y = "GeneID", all.x=F, all.y=F)
resNormdata_Sym <- merge(resNormdata, GeneID_Symbol, by.x = "GeneID",
                         by.y = "GeneID", all.x=F, all.y=F)
write.csv(resdata_Sym, file="hADSCABCDE_Symbols_HxNx_DESeq2-originalresults.csv")
write.csv(resNormdata_Sym, file="hADSCABCDE_Symbols_HxNx_DESeq2-LFCshrunkresults.csv")
```

Plot volcano plot

```
resNormdata_Sym <- read.csv(file = "hADSCABCDE_Symbols_HxNx_DESeq2-LFCshrunkresults.csv",
                            row.names = 1)
resNormdata_Sym$pvalue <- resNormdata_Sym$pvalue + 1e-298
resNormdata_Sym$padj <- resNormdata_Sym$padj + 1e-294
resNormPlot <- data.frame(resNormdata_Sym,
                          Sig=ifelse(resNormdata_Sym$padj<=.05
                                     & abs(resNormdata_Sym$log2FoldChange)>=2,
                                     "P-adj<0.05", "NS"))
resNormPlot_Sig <- subset(resNormPlot, abs(resNormdata_Sym$log2FoldChange)>=2
                          & resNormdata_Sym$padj<=.05, na.rm = F)
resNormPlot_Sig <- resNormPlot_Sig[order(resNormPlot_Sig$log2FoldChange),]
Ligands <- read.delim(file = 'LR_L_R.csv', header = T, sep = ",")
resNormPlot_Sig_L <- merge(resNormPlot_Sig, Ligands, by.x = "GeneSym",
                           by.y = "Ligand.ApprovedSymbol", all.x=F, all.y=F)
resNormPlot_Sig_L <- resNormPlot_Sig_L[order(resNormPlot_Sig_L$log2FoldChange),]
write.csv(resNormPlot_Sig,
          file="hADSCABCDE_Symbols_HxNx_DESeq2-LFCshrunkresults_Sig.csv")
#This gives a list of significant targets for GO
write.csv(resNormPlot_Sig_L,
          file="hADSCABCDE_Symbols_HxNx_DESeq2-LFCshrunkresults_Sig_Ligands.csv")
#This gives a list of significant targets that are ligands
```

Subset based on padj<=0.05 & L2FC |1.5|

Subset based on padj<=0.05 & L2FC |1|

Using ggplot2 (+ ggrepel), plot L2FC

```
VolcPlot = ggplot(resNormPlot, aes(x=log2FoldChange, y=-log10(padj))) +
  geom_point(aes(col=Sig), size=0.1) +
  scale_color_manual(values=c("grey50", "red"))+
  geom_hline(aes(yintercept=-log(0.05)), col="green4", linetype="dashed", size = 0.7) +
  geom_vline(aes(xintercept = -2), col= "green4", linetype="dashed", size = 0.7) +
  geom_vline(aes(xintercept = 2), col= "green4", linetype="dashed", size = 0.7) +
  coord_cartesian(xlim=c(-14,14), ylim=c(0,300)) +
  theme_light() +
  theme(legend.position = "none", axis.text=element_text(size=10)) +
  labs( x = "Log2 Fold Change", y = "-Log10 adj-P")
VolcPlot + geom_text_repel(data = head(resNormPlot_Sig, 20), aes(label=GeneSym),
                           size = 3, nudge_x = -3, direction = "y",
                           segment.size = 0.2, segment.colour = "grey25", hjust = 0.5) +
  geom_text_repel(data = tail(resNormPlot_Sig, 20), aes(label=GeneSym),
                  size = 3,nudge_x = 3, direction = "y",
                           segment.size = 0.2, segment.colour = "grey25", hjust = 0.5) +
  ggtitle("DEG - hADSC-ABCDE in Hx vs Nx") +
  theme(plot.title = element_text(hjust = 0.5)) +
  scale_x_continuous(
    breaks = c(-14, -12, -10, -8, -6, -4, -2, 0, 2, 4, 6, 8, 10, 12, 14),
    limits = c(-14, 14))
```

```r
ggsave("hADSCABCDEVolc2FC.pdf", width = 8, height = 6)
ggsave("hADSCABCDEVolc2FC.tiff", width = 8, height = 6)
```

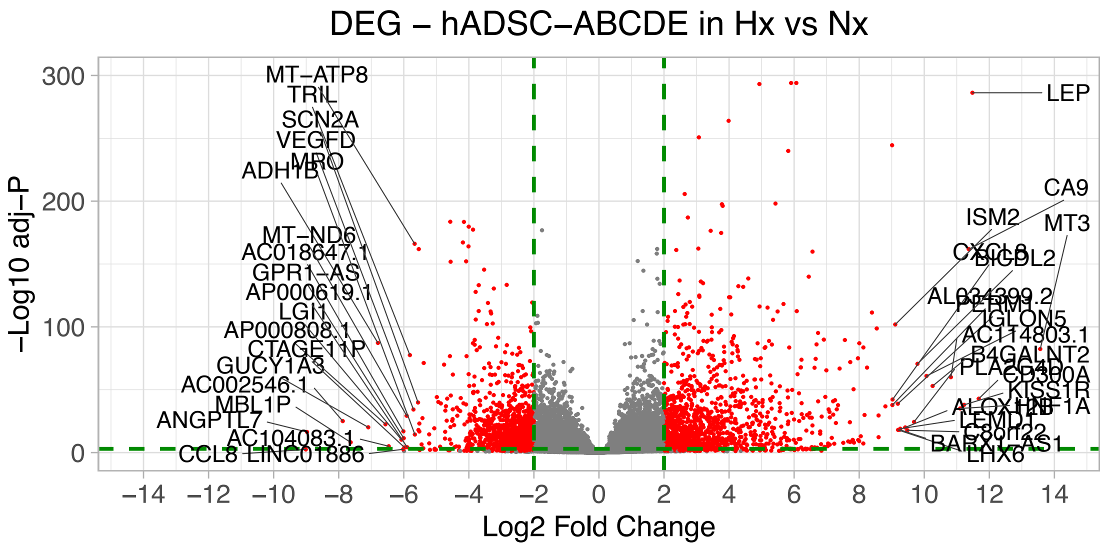

Data quality assessment - sample clustering and visualization

Heatmap of hADSC Hx vs Nx - Transcript variant

```r
rld <- rlog(dds, blind = F)
vsd <- vst(dds, blind = F)
ntd <- normTransform(dds)
library("vsn")
select <- order(rowMeans(counts(dds,normalized=T)),
                decreasing=T)[1:50]
df <- as.data.frame(colData(dds)[,c("conditions", "celltypes")])
rownames(df) <- colnames(ntd)
colnames(df) <- c("conditions", "celltypes")
pheatmap(assay(vsd)[select,], cluster_rows=F, fontsize = 1,
         show_rownames=F, cluster_cols=T, annotation_col=df)
```

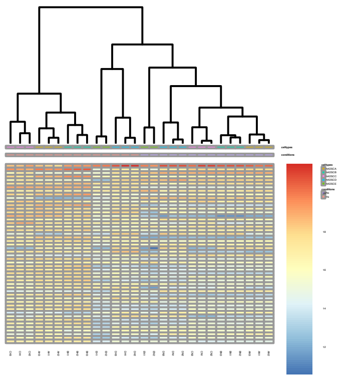

Replot with final configurations

```
select <- order(rowMeans(counts(dds,normalized=T)), decreasing=T)[1:50]
topVarGenes <- head(order(-rowVars(assay(rld))),50)
TVGmat <- assay(vsd)[ topVarGenes,]
TVGmat <- TVGmat - rowMeans(TVGmat)
df <- as.data.frame(colData(dds)[,c("conditions", "celltypes")])
pheatmap(TVGmat, border_color = "grey30", cluster_rows=TRUE,
         show_rownames=TRUE, fontsize_row = 10,
         cluster_cols=TRUE, annotation_col=df, fontsize = 10,
         main = "Top 50 Transcript Variants in hADSC-ABCDE in Hx vs Nx",
         filename = "Heatmap_hADSCABCDE_HxNx_TranscriptVariant.tiff",
         width = 10, height = 12)
```

Heatmap of hADSC Hx vs Nx - sample-to-sample distance

```
sampleDists <- dist(t(assay(vsd)))
library("RColorBrewer")
sampleDistMatrix <- as.matrix(sampleDists)
rownames(sampleDistMatrix) <- paste(vsd$celltypes, vsd$conditions, sep="_")
colnames(sampleDistMatrix) <- NULL
colors <- colorRampPalette( rev(brewer.pal(9, "Blues")) )(250)
pheatmap(sampleDistMatrix, border_color = "grey30",
         clustering_distance_rows=sampleDists,
         clustering_distance_cols=sampleDists,
         col=colors, fontsize = 10, fontsize_row = 15,
         main = "Sample-to-sample Distances",
         filename = "Heatmap_stsdist_hADSCABCDE_HxNx.pdf",
         width = 8, height = 7)
```

PCA plot of samples

```
pcaData <- plotPCA(vsd, intgroup=c("conditions", "celltypes"), returnData=T)
percentVar <- round(100 * attr(pcaData, "percentVar"))
ggplot(pcaData, aes(PC1, PC2, shape=conditions, color=celltypes)) +
  geom_point(size=4) +
  xlab(paste0("PC1: ",percentVar[1],"% variance")) +
  ylab(paste0("PC2: ",percentVar[2],"% variance")) +
  theme_bw() +
  ggtitle("PCA of hADSC-ABCDE in Hx vs Nx") +
  theme(plot.title = element_text(hjust = 0.5)) +
  coord_fixed()
ggsave("PCA_hADSCABCDE_HxNx.pdf", width = 6, height = 4)
```

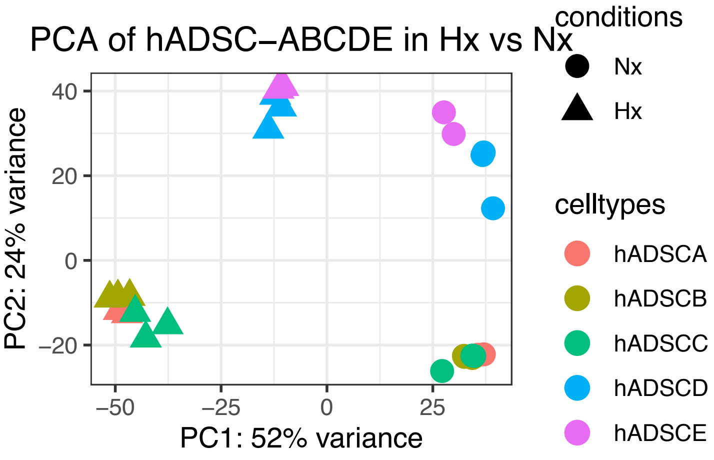

Selecting for Housekeeping genes

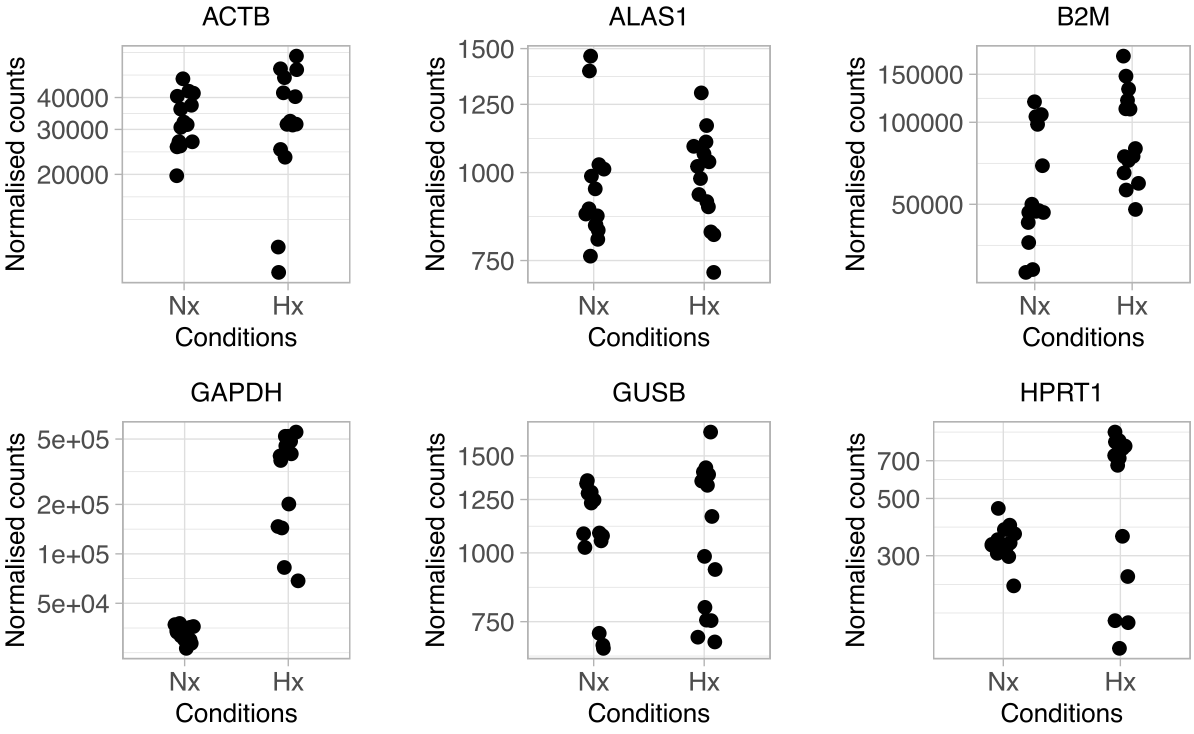

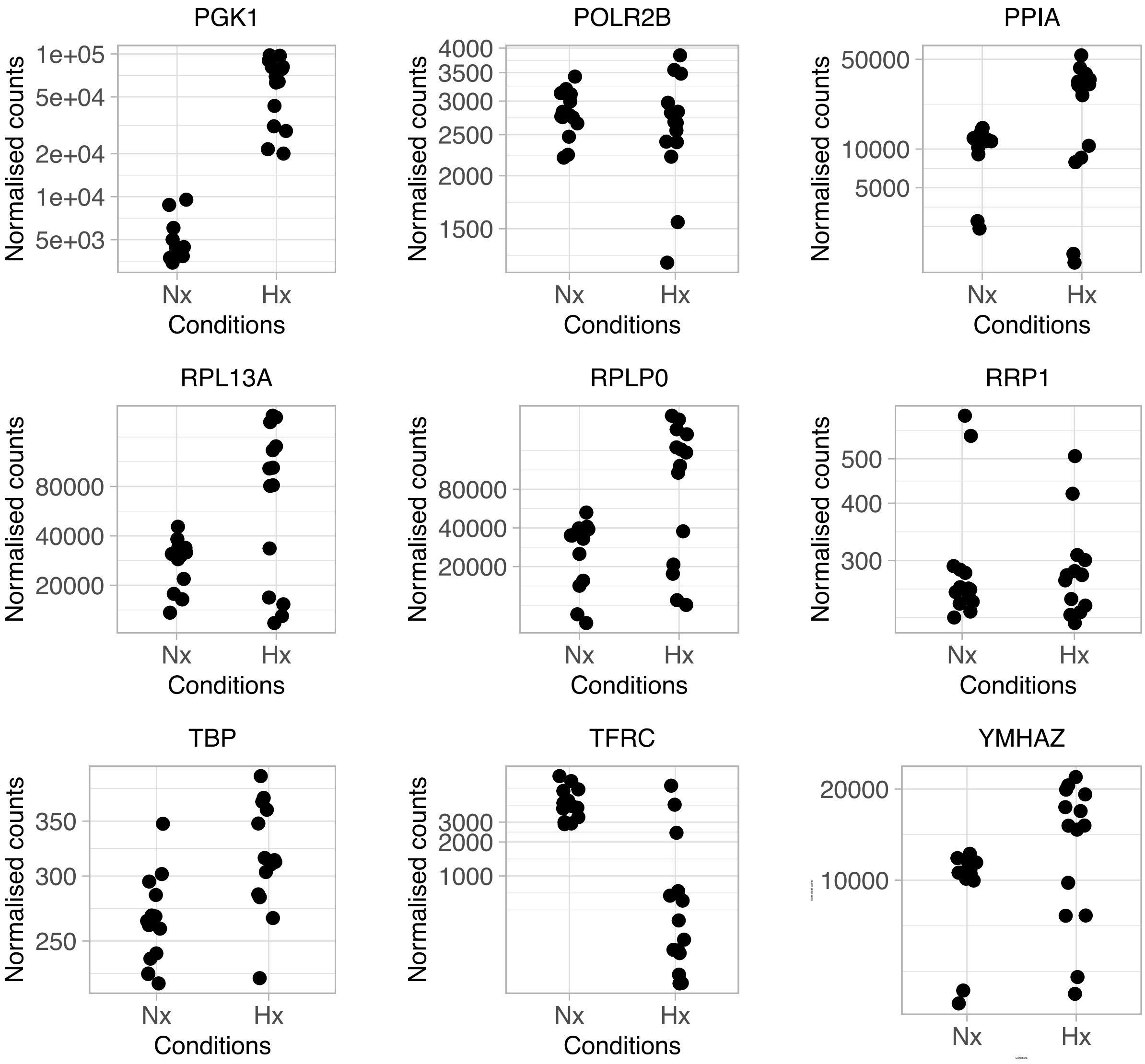

Normalise raw counts to TPM

```
hADSCA_HxNx = read.delim(file = 'Combined_hADSCA.txt', header = T, sep = "")
hADSCB_HxNx = read.delim(file = 'Combined_hADSCB.txt', header = T, sep = "")
hADSCC_HxNx = read.delim(file = 'Combined_hADSCC.txt', header = T, sep = "")
hADSCD_HxNx = read.delim(file = 'Combined_hADSCD.txt', header = T, sep = "")
hADSCE_HxNx = read.delim(file = 'Combined_hADSCE.txt', header = T, sep = "")
#hADSCA
hADSCA_HxNx_TPM <- hADSCA_HxNx[c("Geneid", "GeneSym", "Length", "AH1", "AH2", "AH3",
                                 "AN1", "AN2", "AN3")]
hADSCA_HxNx_TPM$RPKAH1 <- (hADSCA_HxNx_TPM$AH1)/ ((hADSCA_HxNx_TPM$Length)/1000)
totalRPKAH1 <- (sum(hADSCA_HxNx_TPM$AH1))/1000000
hADSCA_HxNx_TPM$TPMAH1 <- (hADSCA_HxNx_TPM$RPKAH1)/ totalRPKAH1
hADSCA_HxNx_TPM$RPKAH2 <- (hADSCA_HxNx_TPM$AH2)/ ((hADSCA_HxNx_TPM$Length)/1000)
totalRPKAH2 <- (sum(hADSCA_HxNx_TPM$AH2))/1000000
hADSCA_HxNx_TPM$TPMAH2 <- (hADSCA_HxNx_TPM$RPKAH2)/ totalRPKAH2
hADSCA_HxNx_TPM$RPKAH3 <- (hADSCA_HxNx_TPM$AH3)/ ((hADSCA_HxNx_TPM$Length)/1000)
```

```
totalRPKAH3 <- (sum(hADSCA_HxNx_TPM$AH3))/1000000
hADSCA_HxNx_TPM$TPMAH3 <- (hADSCA_HxNx_TPM$RPKAH3)/ totalRPKAH3
hADSCA_HxNx_TPM$RPKAN1 <- (hADSCA_HxNx_TPM$AN1)/ ((hADSCA_HxNx_TPM$Length)/1000)
totalRPKAN1 <- (sum(hADSCA_HxNx_TPM$AN1))/1000000
hADSCA_HxNx_TPM$TPMAN1 <- (hADSCA_HxNx_TPM$RPKAN1)/ totalRPKAN1
hADSCA_HxNx_TPM$RPKAN2 <- (hADSCA_HxNx_TPM$AN2)/ ((hADSCA_HxNx_TPM$Length)/1000)
totalRPKAN2 <- (sum(hADSCA_HxNx_TPM$AN2))/1000000
hADSCA_HxNx_TPM$TPMAN2 <- (hADSCA_HxNx_TPM$RPKAN2)/ totalRPKAN2
hADSCA_HxNx_TPM$RPKAN3 <- (hADSCA_HxNx_TPM$AN3)/ ((hADSCA_HxNx_TPM$Length)/1000)
totalRPKAN3 <- (sum(hADSCA_HxNx_TPM$AN3))/1000000
hADSCA_HxNx_TPM$TPMAN3 <- (hADSCA_HxNx_TPM$RPKAN3)/ totalRPKAN3
hADSCA_HxNx_onlyTPM <- hADSCA_HxNx_TPM[c("Geneid", "GeneSym",
                                         "TPMAH1", "TPMAH2", "TPMAH3",
                                         "TPMAN1", "TPMAN2", "TPMAN3")]
write.table(hADSCA_HxNx_onlyTPM, file = "Combined_hADSCA_onlyTPM.txt", quote = F,
            sep = "\t", na = "NA", row.names = F, col.names = T)
```

And for hADSC-B,C,D,E

```
hADSCA_HxNx_onlyTPM <- hADSCA_HxNx_onlyTPM[ ,c("Geneid",
                                         "TPMAH1", "TPMAH2", "TPMAH3",
                                         "TPMAN1", "TPMAN2", "TPMAN3")]
hADSCB_HxNx_onlyTPM <- hADSCB_HxNx_onlyTPM[ ,c("Geneid",
                                         "TPMBH1", "TPMBH2", "TPMBH3",
                                         "TPMBN1", "TPMBN2", "TPMBN3")]
hADSCC_HxNx_onlyTPM <- hADSCC_HxNx_onlyTPM[ ,c("Geneid",
                                         "TPMCH1", "TPMCH2", "TPMCH3",
                                         "TPMCN1", "TPMCN2", "TPMCN3")]
hADSCD_HxNx_onlyTPM <- hADSCD_HxNx_onlyTPM[ ,c("Geneid",
                                         "TPMDH1", "TPMDH2", "TPMDH3",
                                         "TPMDN1", "TPMDN2", "TPMDN3")]
ABCDE_HxNx_onlyTPM <- merge(hADSCD_HxNx_onlyTPM, hADSCE_HxNx_onlyTPM,
                            by.x = "Geneid", by.y = "Geneid", all.x=T, all.y=T)
ABCDE_HxNx_onlyTPM <- merge(hADSCC_HxNx_onlyTPM, ABCDE_HxNx_onlyTPM,
                            by.x = "Geneid", by.y = "Geneid", all.x=T, all.y=T)
ABCDE_HxNx_onlyTPM <- merge(hADSCB_HxNx_onlyTPM, ABCDE_HxNx_onlyTPM,
                            by.x = "Geneid", by.y = "Geneid", all.x=T, all.y=T)
ABCDE_HxNx_onlyTPM <- merge(hADSCA_HxNx_onlyTPM, ABCDE_HxNx_onlyTPM,
                            by.x = "Geneid", by.y = "Geneid", all.x=T, all.y=T)
write.table(ABCDE_HxNx_onlyTPM, file = "Combined_ABCDE_onlyTPM.txt", quote = F,
            sep = "\t",na = "NA", row.names = F, col.names = T)
```

Extract L2FC and TPM values for selected HKG

```
SelectedHKG  <- as.matrix(read.csv(file = 'SelectedHKG.csv', sep=",", header = T))
hADSCABCDE_DESeq2  <- as.matrix(
  read.csv(file = 'hADSCABCDE_Symbols_HxNx_DESeq2-LFCshrunkresults.csv',
           sep=",", header = T, row.names = 1))
SelectedHKG <- merge(hADSCABCDE_DESeq2, SelectedHKG,
                     by.x = "GeneSym", by.y = "Gene_Sym", all.x=F, all.y=T)
SelectedHKG <- merge(ABCDE_HxNx_onlyTPM, SelectedHKG,
                     by.x = "Geneid", by.y = "GeneID", all.x=F, all.y=T)
write.csv(SelectedHKG, file="SelectedHKG_RNAseqData.csv")
```
