# Supplementary material for: Using RNA-seq to identify suitable housekeeping genes for hypoxia studies in human adipose-derived stem cells
Source: BMC Mol Cell Biol. 2023 Apr 17;24:16. doi: 10.1186/s12860-023-00475-4 (PMC10108514; doi:10.1186/s12860-023-00475-4)
Supplement: Supplementary file 5 — Additional file 5. Summary of hADSC donor information and cell characterisation used in this study. [file 12860_2023_475_MOESM5_ESM.pdf]

Additional File 5. Summary of hADSC donor information and cell characterisation used in this study

Cell ID	hADSC-A	hADSC-B	hADSC-C	hADSC-D	hADSC-E
Supplier	Lonza	Lonza	Zenbio	Zenbio	Lonza
Lot number	0000669429	0000605220	ASC120116E	ASC072709	0000440549
Age	42	22	52	39	44
Sex	Female	Male	Female	Female	Female
BMI	32	32	26.5	38	33
Race	Hispanic	Other	Caucasian	Unknown	African American
Tissue	Unknown	Unknown	Abdomen	Abdomen/Hip	Unknown
Virus	-ve	-ve	-ve	-ve	-ve
Microbial	-ve	-ve	-ve	-ve	-ve
Passage frozen	1	1	2	2	1
Cell viability	93%	91%	N/A	N/A	73%
Cell count (viable cells/ml)	1.968×10^6	2.32×10^6	$\geq 1.0 \times 10^6$	$\geq 1.0 \times 10^6$	1.415×10^6
Seeding efficiency	80%	79%	N/A	N/A	58%
Doubling time (h)	22	21	N/A	N/A	22
CD13<br>CD29<br>CD44<br>CD73<br>CD90<br>CD105<br>CD166<br>CD14<br>CD31<br>CD45<br>CD34	≥ 90%<br>≥ 90%<br>≥ 90%<br>≥ 90%<br>≥ 90%<br>≥ 90%<br>≥ 90%<br>≤ 5%<br>≤ 5%<br>≤ 5%<br>0%	≥ 90%<br>≥ 90%<br>≥ 90%<br>≥ 90%<br>≥ 90%<br>≥ 90%<br>≥ 90%<br>≤ 5%<br>≤ 5%<br>≤ 5%<br>≤ 5%	N/A<br>N/A<br>99.4%<br>N/A<br>N/A<br>99.8%<br>N/A<br>N/A<br>0%<br>0.6%<br>N/A	N/A<br>N/A<br>91.3%<br>N/A<br>N/A<br>94%<br>N/A<br>N/A<br>0%<br>0.3%<br>N/A	≥ 90%<br>≥ 90%<br>≥ 90%<br>≥ 90%<br>≥ 90%<br>≥ 90%<br>≥ 90%<br>≤ 5%<br>≤ 5%<br>≤ 5%<br>3%
Adipogenic<br>Osteogenic<br>Chondrogenic	N/A	N/A	+ve<br>+ve<br>+ve	+ve<br>+ve<br>+ve	N/A
