# Supplementary material for: Using RNA-seq to identify suitable housekeeping genes for hypoxia studies in human adipose-derived stem cells
Source: BMC Mol Cell Biol. 2023 Apr 17;24:16. doi: 10.1186/s12860-023-00475-4 (PMC10108514; doi:10.1186/s12860-023-00475-4)
Supplement: Supplementary file 6 — Additional file 6. Quality control and qualification results of RNA extracted from hADSC. [file 12860_2023_475_MOESM6_ESM.pdf]

Additional File 6. Quality control and quantification results of RNA extracted from hADSC

Batch	Sample Name	Peak count	Total Conc. (ng/ul)	RNA Area	rRNA Area Ratio [28S/18S]	rRNA Height Ratio [28S/18S]	rRNA Fast Area Ratio	RNA Quality Score	5S Area	5S % Total	18S Area	18S % Total	28S Area	28S % Total
1	EH1	-	58.00	82.60	2.30	-	-	10.0	-	-	14.70	0.18	33.50	0.41
1	EH2	-	88.00	126.50	2.00	-	-	10.0	-	-	24.20	0.19	47.90	0.38
1	EN1	-	201.00	287.50	2.00	-	-	10.0	-	-	58.80	0.21	115.20	0.40
1	EN2	-	128.00	182.60	2.30	-	-	10.0	-	-	37.50	0.21	86.90	0.48
1	Ladder1	-	150.00	160.10	-	-	-	-	-	-	172.70	0.24	378.60	0.52
2	AN1	24	438.63	493.20	3.45	1.47	0.03	9.7	2.69	0.01	65.13	0.13	224.65	0.46
2	AN2	20	372.80	419.51	3.53	1.57	0.03	9.7	1.89	0.00	53.27	0.13	187.79	0.45
2	AN3	14	190.25	213.85	3.92	1.80	0.03	10.0	0.99	0.01	27.65	0.13	108.26	0.51
2	AH1	13	335.42	379.14	2.46	1.16	0.04	9.7	3.53	0.01	70.33	0.19	173.05	0.46
2	AH2	12	201.46	228.33	2.11	0.94	0.04	9.5	2.17	0.01	46.47	0.20	98.08	0.43
2	AH3	9	114.56	131.62	3.02	1.29	0.04	9.9	0.00	0.00	22.04	0.17	66.47	0.51
2	BN1	24	397.02	460.44	3.45	1.39	0.04	9.6	4.13	0.01	61.52	0.13	212.06	0.46
2	BN2	17	353.52	408.47	3.04	1.44	0.04	9.6	4.16	0.01	59.59	0.15	181.22	0.44
2	BN3	14	221.35	258.22	3.24	1.57	0.05	10.0	2.60	0.01	42.37	0.16	137.24	0.53
2	BH1	17	248.78	286.48	2.43	1.19	0.04	9.5	1.37	0.01	49.53	0.17	120.30	0.42
2	BH2	11	172.04	193.78	2.42	1.14	0.04	9.6	0.59	0.00	35.50	0.18	85.82	0.44
2	BH3	11	147.93	172.70	2.85	1.04	0.05	9.9	1.03	0.01	32.20	0.19	91.64	0.53
2	CN1	16	475.88	556.58	2.96	1.43	0.03	9.6	3.23	0.01	79.59	0.14	235.80	0.42
2	CN2	25	509.46	598.71	3.29	1.33	0.03	[9.6]	3.40	0.01	83.13	0.14	273.30	0.46
2	CN3	14	260.32	302.02	3.90	1.66	0.03	10.0	1.57	0.01	46.06	0.15	179.68	0.60
2	CH1	11	232.80	271.91	2.32	1.09	0.04	9.7	2.97	0.01	54.50	0.20	126.51	0.47
2	CH2	13	252.65	294.59	2.39	1.11	0.06	9.7	3.58	0.01	58.49	0.20	139.93	0.48
2	CH3	15	258.26	301.10	2.28	1.12	0.04	9.7	1.81	0.01	58.72	0.20	134.03	0.45
2	DN1	11	115.56	135.51	3.52	1.50	0.06	9.8	1.17	0.01	19.17	0.14	67.50	0.50
2	DN2	14	108.09	126.52	3.21	1.63	0.06	9.9	1.14	0.01	19.40	0.15	62.29	0.49
2	DN3	13	176.47	205.03	3.11	1.39	0.06	9.6	2.00	0.01	30.80	0.15	95.82	0.47
2	DH1	18	125.21	147.58	3.32	1.78	0.04	9.5	0.34	0.00	17.60	0.12	58.49	0.40
2	DH2	12	104.17	119.30	3.18	1.66	0.05	9.7	0.53	0.00	16.71	0.14	53.15	0.45
2	DH3	12	152.23	179.45	2.83	1.51	0.05	9.3	0.25	0.00	23.35	0.13	66.18	0.37
2	Ladder2	-	480.00	539.39	-	-	-	-	-	-	-	-	-	-
