# Supplementary material for: Using RNA-seq to identify suitable housekeeping genes for hypoxia studies in human adipose-derived stem cells
Source: BMC Mol Cell Biol. 2023 Apr 17;24:16. doi: 10.1186/s12860-023-00475-4 (PMC10108514; doi:10.1186/s12860-023-00475-4)
Supplement: Supplementary file 7 — Additional file 7. RNAseq processing efficiencies represented as percentage counts of original sequenced reads [file 12860_2023_475_MOESM7_ESM.pdf]

Additional File 7. RNAseq processing efficiencies represented as percentage counts of original sequenced reads

File ID	AH1		AH2		AH3		AN1		AN2		AN3	
Samples	hADSCA_Hx1		hADSCA_Hx2		hADSCA_Hx3		hADSCA_Nx1		hADSCA_Nx2		hADSCA_Nx3	
Lanes	1	2	1	2	1	2	1	2	1	2	1	2
Raw reads	16,865,742	16,805,837	19,563,460	19,355,715	18,147,753	18,041,931	19,189,651	18,894,401	16,131,994	15,987,693	18,403,287	18,152,453
%	100%	100%	100%	100%	100%	100%	100%	100%	100%	100%	100%	100%
Trimmed	16,770,348	16,711,988	19,443,379	19,237,780	18,034,438	17,931,230	19,074,701	18,783,458	16,034,955	15,892,463	18,282,496	18,036,012
%	99.43%	99.44%	99.39%	99.39%	99.38%	99.39%	99.40%	99.41%	99.40%	99.40%	99.34%	99.36%
Mapped	16,647,019	16,589,377	19,298,992	19,093,980	17,901,897	17,799,331	18,928,446	18,638,343	15,916,774	15,774,758	18,135,141	17,890,321
%	99.26%	99.27%	99.26%	99.25%	99.27%	99.26%	99.23%	99.23%	99.26%	99.26%	99.19%	99.19%
Combined	33,236,396		38,392,972		35,701,228		37,566,789		31,691,532		36,025,462	
%	99.27%		99.25%		99.26%		99.23%		99.26%		99.19%	
Filtered	29,561,768		34,313,212		31,416,984		35,424,597		29,903,302		33,702,591	
%	88.94%		89.37%		88.00%		94.30%		94.36%		93.55%	
Counted	28,286,728		32,856,271		30,048,517		33,564,732		28,343,297		31,786,994	
%	95.69%		95.75%		95.64%		94.75%		94.78%		94.32%	

File ID	BH1		BH2		BH3		BN1		BN2		BN3	
Samples	hADSCB_Hx1		hADSCB_Hx2		hADSCB_Hx3		hADSCB_Nx1		hADSCB_Nx2		hADSCB_Nx3	
Lanes	1	2	1	2	1	2	1	2	1	2	1	2
Raw reads	20,586,863	20,366,816	15,799,509	15,652,593	23,442,687	23,324,448	20,156,333	19,978,469	18,961,761	18,648,201	18,907,731	18,765,141
%	100%	100%	100%	100%	100%	100%	100%	100%	100%	100%	100%	100%
Trimmed	20,458,101	20,240,126	15,693,487	15,548,634	23,313,101	23,196,830	20,030,576	19,856,130	18,841,306	18,530,993	18,782,597	18,642,378
%	99.37%	99.38%	99.33%	99.34%	99.45%	99.45%	99.38%	99.39%	99.36%	99.37%	99.34%	99.35%
Mapped	20,299,327	20,083,029	15,567,548	15,424,402	23,148,506	23,032,524	19,875,590	19,701,417	18,698,875	18,389,619	18,632,726	18,492,482
%	99.22%	99.22%	99.20%	99.20%	99.29%	99.29%	99.23%	99.22%	99.24%	99.24%	99.20%	99.20%
Combined	40,382,356		30,991,950		46,181,030		39,577,007		37,088,494		37,125,208	
%	99.22%		99.20%		99.29%		99.22%		99.24%		99.20%	
Filtered	33,524,128		26,392,336		32,020,313		37,252,314		34,908,324		34,722,969	
%	83.02%		85.16%		69.34%		94.13%		94.12%		93.53%	
Counted	32,017,847		25,175,882		30,479,023		35,409,589		33,237,275		32,835,703	
%	95.51%		95.39%		95.19%		95.05%		95.21%		94.56%	

File ID	CH1		CH2		CH3		CN1		CN2		CN3	
Samples	hADSCC_Hx1		hADSCC_Hx2		hADSCC_Hx3		hADSCC_Nx1		hADSCC_Nx2		hADSCC_Nx3	
Lanes	1	2	1	2	1	2	1	2	1	2	1	2
Raw reads	18,563,508	18,424,482	19,109,693	18,982,963	18,638,507	18,348,910	32,640,063	32,454,383	27,982,041	27,634,981	17,003,999	16,787,483
%	100%	100%	100%	100%	100%	100%	100%	100%	100%	100%	100%	100%
Trimmed	18,444,978	18,308,143	18,994,622	18,869,744	18,518,278	18,232,476	32,445,921	32,264,022	27,812,981	27,469,539	16,898,713	16,684,567
%	99.36%	99.37%	99.40%	99.40%	99.35%	99.37%	99.41%	99.41%	99.40%	99.40%	99.38%	99.39%
Mapped	18,301,873	18,163,610	18,854,505	18,730,820	18,376,615	18,092,841	32,205,955	32,025,634	27,609,762	27,268,970	16,777,361	16,564,362
%	99.22%	99.21%	99.26%	99.26%	99.24%	99.23%	99.26%	99.26%	99.27%	99.27%	99.28%	99.28%
Combined	36,465,483		37,585,325		36,469,456		64,231,589		54,878,732		33,341,723	
%	99.22%		99.26%		99.23%		99.26%		99.27%		99.28%	
Filtered	32,299,421		33,498,328		31,483,104		60,393,267		51,591,449		30,785,185	
%	88.58%		89.13%		86.33%		94.02%		94.01%		92.33%	
Counted	30,899,670		32,077,009		30,025,493		57,115,543		48,805,795		29,294,406	
%	95.67%		95.76%		95.37%		94.57%		94.60%		95.16%	

File ID	DH1		DH2		DH3		DN1		DN2		DN3	
Samples	hADSCD_Hx1		hADSCD_Hx2		hADSCD_Hx3		hADSCD_Nx1		hADSCD_Nx2		hADSCD_Nx3	
Lanes	1	2	1	2	1	2	1	2	1	2	1	2
Raw reads	24,968,883	24,668,407	22,666,792	22,426,689	18,309,259	18,090,563	23,839,312	23,610,769	20,754,751	20,399,319	28,866,793	28,575,716
%	100%	100%	100%	100%	100%	100%	100%	100%	100%	100%	100%	100%
Trimmed	24,819,392	24,523,168	22,522,966	22,285,951	18,198,483	17,982,458	23,706,055	23,479,424	20,628,190	20,276,877	28,694,424	28,406,806
%	99.40%	99.41%	99.37%	99.37%	99.39%	99.40%	99.44%	99.44%	99.39%	99.40%	99.40%	99.41%
Mapped	24,596,288	24,301,590	22,311,878	22,074,963	18,022,134	17,808,003	23,506,074	23,279,959	20,450,795	20,102,497	28,449,284	28,163,908
%	99.10%	99.10%	99.06%	99.05%	99.03%	99.03%	99.16%	99.15%	99.14%	99.14%	99.15%	99.14%
Combined	48,897,878		44,386,841		35,830,137		46,786,033		40,553,292		56,613,192	
%	99.10%		99.06%		99.03%		99.15%		99.14%		99.15%	
Filtered	46,601,967		42,296,543		33,396,746		44,337,464		38,443,980		53,595,945	
%	95.30%		95.29%		93.21%		94.77%		94.80%		94.67%	
Counted	43,265,853		39,277,707		31,757,373		41,437,732		36,059,418		50,905,484	
%	92.84%		92.86%		95.09%		93.46%		93.80%		94.98%	

File ID	B9	B10	B11	B12	B19	B20	B21
Samples	hADSCE_Hx1	hADSCE_Hx2	hADSCE_Nx1	hADSCE_Nx2	hTMk 1	hTMk 2	hTMk 3
Lanes	1	1	1	1	1	1	1
Raw reads	9,763,834	10,445,533	8,462,302	10,295,620	7,730,453	9,100,826	8,771,665
%	100%	100%	100%	100%	100%	100%	100%
Trimmed	9,482,475	10,310,402	8,258,382	10,169,650	7,637,943	8,980,691	8,649,143
%	97.12%	98.71%	97.59%	98.78%	98.80%	98.68%	98.60%
Mapped	9,260,753	10,081,910	8,099,028	9,912,166	7,499,093	8,823,812	8,499,844
%	97.66%	97.78%	98.07%	97.47%	98.18%	98.25%	98.27%
Filtered	8,453,498	9,187,612	7,382,408	9,052,920	6,219,578	7,318,955	7,142,776
%	91.28%	91.13%	91.15%	91.33%	82.94%	82.95%	84.03%
Counted	8,025,156	8,737,504	7,007,689	8,575,136	5,899,104	6,946,373	6,779,418
%	94.93%	95.10%	94.92%	94.72%	94.85%	94.91%	94.91%
